# Novel α-L-Fucosidases from a Soil Metagenome for Production of Fucosylated Human Milk Oligosaccharides

**DOI:** 10.1371/journal.pone.0147438

**Published:** 2016-01-22

**Authors:** Mateusz Lezyk, Carsten Jers, Louise Kjaerulff, Charlotte H. Gotfredsen, Maria D. Mikkelsen, Jørn D. Mikkelsen

**Affiliations:** 1 Center for BioProcess Engineering, Department of Chemical and Biochemical Engineering, Technical University of Denmark, Kgs. Lyngby, Denmark; 2 Department of Chemistry, Technical University of Denmark, Kgs. Lyngby, Denmark; 3 Department of Plant and Environmental Sciences, University of Copenhagen, Copenhagen, Denmark; Dong-A University, REPUBLIC OF KOREA

## Abstract

This paper describes the discovery of novel α-L-fucosidases and evaluation of their potential to catalyse the transglycosylation reaction leading to production of fucosylated human milk oligosaccharides. Seven novel α-L-fucosidase-encoding genes were identified by functional screening of a soil-derived metagenome library and expressed in *E*. *coli* as recombinant 6xHis-tagged proteins. All seven fucosidases belong to glycosyl hydrolase family 29 (GH 29). Six of the seven α-L-fucosidases were substrate-inhibited, moderately thermostable and most hydrolytically active in the pH range 6–7, when tested with *para*-nitrophenyl-α-L-fucopyranoside (pNP-Fuc) as the substrate. In contrast, one fucosidase (Mfuc6) exhibited a high pH optimum and an unusual sigmoidal kinetics towards pNP-Fuc substrate. When tested for trans-fucosylation activity using pNP-Fuc as donor, most of the enzymes were able to transfer fucose to pNP-Fuc (self-condensation) or to lactose. With the α-L-fucosidase from *Thermotoga maritima* and the metagenome-derived Mfuc5, different fucosyllactose variants including the principal fucosylated HMO 2’-fucosyllactose were synthesised in yields of up to ~6.4%. Mfuc5 was able to release fucose from xyloglucan and could also use it as a fucosyl-donor for synthesis of fucosyllactose. This is the first study describing the use of glycosyl hydrolases for the synthesis of genuine fucosylated human milk oligosaccharides.

## Introduction

L-Fucose (6-deoxy-L-galactose) plays a significant role in a wide range of biological processes such as receptor signaling, inflammation, metastasis and disease (fucosidosis) in mammals [1, 2]. L-Fucose is also an important constituent in human milk oligosaccharides (HMOs) where 50–80% of the HMOs are fucosylated [3]. Three of the more abundant fucosylated HMOs, 2’-fucosyllactose (2’-FL), 3-fucosyllactose (3-FL) and lactodifucotetraose have been shown to exhibit a prebiotic effect [4]. Besides being prebiotic, 2’-FL and 2’-fucosylated oligosaccharides prevent binding of the pathogen *Campylobacter jejuni* to the intestinal mucosa and thus reduce incidents of diarrhea [5,6]. Recently, it was shown that 2’-FL and 3-FL also regulate gut motility possibly via a specific interaction with tissue receptors [7].

Due to the functional properties of fucosylated HMOs, there is an increasing interest in development of processes for synthesis of these molecules. There are several examples of, to our knowledge exclusively, retaining glycosyl hydrolases that can catalyse trans-glycosylation reactions, i.e. transfer a sugar from a donor molecule to acceptors different than water [8]. α-L-fucosidases are glycosyl hydrolases that cleave fucosidic bonds in oligosaccharides and glycoconjugates. According to the CAZY database [9] all α-L-fucosidases hydrolyzing fucosyl derivatives with retention of the anomeric configuration belong to the GH 29 family, whereas inverting α-L-fucosidases are classified in family GH 95. The retaining GH 29 α-L-fucosidases utilize a classical Koshland double displacement mechanism in which an aspartate (Asp) residue is used as the catalytic nucleophile [10,11].

Several α-L-fucosidases, all belonging to the GH 29 family, have been shown to catalyse trans-fucosylation using *para-*nitrophenyl-α-L-fucopyranoside (pNP-Fuc) as fucosyl donor and in most cases methyl, *para*-nitrophenol (pNP) or allyl derivatives of monosaccharides as acceptor [8]. In one study lactose was used as acceptor, and the α-L-fucosidase from *Alcaligenes* sp. was shown to fucosylate lactose producing specifically the unnatural 3’-FL [12], but so far the HMOs 2’-FL and 3-FL have not been synthesised in trans-fucosylation reactions. 2’-FL and 3-FL have been synthesised using fucosyltransferases and fucosynthases [13], but their use in larger scale synthesis suffers from expensive, and in the case of fucosynthases, unstable donor substrates. The main advantage of α-L-fucosidases is the potential of using cheaper, stable and natural, renewable donor substrates. The natural polymer xyloglucan have been shown to contain fucose [14]. It is therefore of interest to identify enzymes capable of acting on this polymer that in addition can catalyse trans-fucosylation. In that respect, the well-characterised *Thermotoga maritima* α-L-fucosidase is an interesting candidate as it can catalyse trans-fucosylation [15] and has been hypothesised to act on xyloglucan [16].

Functional screening of metagenome libraries for discovery of novel enzymes has gained a lot of attention over the last years [17]. With only a small fraction of the total microbial community being culturable, metagenome libraries provide access to larger diversity in protein sequence space [18]. The most common targets in metagenomic studies have been glycosyl hydrolases and lipases/esterases for which assays suitable for high-throughput screening are available [19]. In contrast, the search for enzymes where the available assay methodology requires e.g. coupled reactions or where the substrate or product lacks a detectable signal, such as trans-glycosylating enzymes, is less trivial [18].

The aim of this study was to identify enzymes that can catalyse trans-fucosylation preferentially using a natural fucose-containing polymer as substrate. In order to provide protein sequence diversity, a soil metagenome was screened for novel α-L-fucosidases. Subsequently, the hydrolytic and trans-fucosylation activity of metagenome-derived α-L-fucosidases, as well as that of *T*. *maritima*, was investigated. The use of α-L-fucosidases to synthesize fucosyllactose (FL) including the human milk fuco-oligosaccharide 2’-FL in yields of ~4–6% using pNP-Fuc was demonstrated and proof of concept for the use of xyloglucan as a fucosyl donor was established.

## Materials and Methods

### Substrates

5-bromo-4-chloro-3-indolyl-α-L-fucopyranoside and pNP-Fuc were purchased from Carbosynth (United Kingdom, Compton). 2’-FL and 3-FL were obtained from Elicityl (Crolles, France). β-lactose was purchased from Sigma-Aldrich (Germany, Steinheim). L-fucose was supplied by DuPont Danisco (Denmark, Copenhagen). Xyloglucan from citrus peel containing 27 mg fucose per g dry matter (determined as described previously [20]) was kindly provided by Hassan Ahmadi Gavlighi from Technical University of Denmark (Denmark, Kgs. Lyngby).

### Strains and plasmids

The strain *E*. *coli* ML297 was used for the maintenance and screening of the constructed metagenome library. It is a Δ*lacZYA* derivative of EPI300™-T1^R^ (Epicentre, USA) that was transformed with the heat-inducible lysis vector pEAS-1a (DualSystems Biotech—Switzerland, Schlieren). This strain was used in order to screen the library for other enzyme classes, including β-galactosidases (to be published elsewhere). *E*. *coli* DH5α was used for sub-cloning and plasmid propagation. *E*. *coli* BL21(DE3) and OverExpress C41 (DE3) strains were used for production of recombinant α-L-fucosidases (Novagen and Lucigen, USA).

### Construction of metagenome library

Metagenome DNA was extracted from 10 g of soil sample (dump soil obtained from RGS 90 A/S at the coordinates 55.629995, 12.536342) using the PowerMax Soil DNA Isolation kit (Mobio Laboratories Inc.–USA, Carlsbad), following the manufacturer’s recommendations. A library of 40–50 kb metagenome DNA fragments in *E*. *coli* was created using the CopyControl Fosmid Library Production kit (Epicentre—USA, Madison). In brief, DNA was end-repaired and high molecular weight (40–50 kb) fragments were purified using 1% low melting point-agarose gel electrophoresis (overnight at 30 V, 4°C). 0.25 μg of metagenome DNA was ligated with 0.5 μg of the linearized fosmid pCC1FOS vector and packaged using replication-deficient phage extract. *E*. *coli* strain ML297 was transfected with the resulting phages and library size was determined by plating serial dilutions on LB agar plates containing 12.5 μg/ml chloramphenicol and 100 μg/ml ampicillin. The *E*. *coli* metagenome DNA library was grown to mid-log phase for 8 h shaking at 30°C in 50 ml LB supplemented with relevant antibiotics, and 2 ml aliquots with 15% glycerol were stored frozen at -80°C. Each frozen stock was subsequently confirmed to have about 50,000 colony forming units (CFU) per ml.

### Screening for α-L-fucosidase-bearing metagenome clones

To screen for novel α-L-fucosidase genes, the *E*. *coli* metagenome library was plated on standard size LB agar plates (10 cm in diameter) supplemented with 34 μg/ml chloramphenicol, 100 μg/ml ampicillin and 100 μg/ml of X-Fuc to a density of approximately 1,000 CFU. In total about 100,000 colonies were cultivated for 3 days at 30°C (to avoid heat-induced lysis) and colonies turning blue were selected. Fosmid material was isolated from liquid cultures of selected clones using FosmidMAX™ DNA Purification Kit (Epicentre). Restriction analyses of fosmids with *Bam*HI and *Pst*I were performed to identify redundant positive clones.

To facilitate sequencing and gene identification, the fosmid DNA was digested with *Bam*HI and further subcloned into *Bam*HI-linearized and dephosphorylated pUC18 vector. Ligated DNA was used to transform electrocompetent cells of *E*. *coli* DH5α and resulting transformants were plated on LB agar plates supplemented with 100 μg/ml ampicillin and 100 μg/ml X-Fuc and incubated overnight at 37°C. Transformants degrading X-Fuc contained fragments of the original fosmid with a functional α-L-fucosidase gene and were selected for further analysis.

### Sequencing, and identification of α-L-fucosidase genes

pUC18-derivatives containing α-L-fucosidase-encoding genes were linearized using *Bam*HI or *Pst*I, purified by gel electrophoresis and sequenced by IonTorrent PGM sequencing using 316 chip (DMAC, Denmark, Lyngby). Sequence reads were trimmed using clc_quality_trim script (CLC BIO, Denmark, Aarhus) and assembled using Ray Meta [21]. BLAST_X_ [22] was used to annotate all contigs. Primers were designed to close gaps between contigs containing truncated α-L-fucosidase-annotated genes at their 5’ or 3’ ends.

### Cloning, expression and purification of α-L-fucosidases

Metagenome-derived genes were PCR-amplified using specific primers (S1 Table) and fosmid DNA as template. PCR products were restricted and inserted in the plasmid pETM10 between *Nco*I and *Kpn*I. A gene encoding the α-L-fucosidase of *T*. *maritima* (Thma) was codon-optimized and synthesized by GeneArt AG (S1 File). The gene was PCR-amplified using relevant primers (S1 Table), restricted and inserted in pETM10 between *Nco*I and *Kpn*I. The resulting plasmids where used to transform *E*. *coli* BL21(DE3) or C41(DE3) strains.

*E*. *coli* BL21(DE3) and C41(DE3) harboring recombinant plasmids were cultured in LB medium shaking at 30°C prior to induction at OD_600_ 0.6 with 0.2 mM or 1 mM IPTG. Expression was continued overnight shaking at 25°C. The cell pellets were harvested by centrifugation and re-suspended in binding buffer (20 mM phosphate-citrate buffer, 500 mM NaCl, 20 mM imidazole, pH 7.4). Cells were lysed by sonication and centrifuged at 20000 g for 30 min. The supernatant was subjected to sterile filtration through a 0.22 μm filter and subsequently purified using an ÄKTA purifier with Ni^2+^-sepharose HisTrap HP column (5 ml, GE healthcare) mounted according to manufacturer’s recommendations. Protein concentrations were determined using the BCA protein assay (Thermo scientific, USA, Waltham) with BSA as the standard.

### Hydrolase activity assays

The pH-dependence for hydrolysis was tested using 0.1 and 1.0 mM pNP-Fuc as substrate at 30°C. The reactions were done in triplicate with the following buffers: 50 mM phosphate-citrate buffer (pH 3–8), 50 mM tricine buffer (pH 8–9), 50 mM glycylglycine buffer (pH 8–9), 50 mM glycine-NaOH buffer (pH 9–10). Reactions with 0.1 mM pNP-Fuc were initiated by addition of enzyme at a final concentration of 0.2 μg/ml Mfuc1, 0.2 μg/ml Mfuc2, 0.6 μg/ml Mfuc3, 0.3 μg/ml Mfuc4, 0.2 μg/ml Mfuc5, 1.0 μg/ml Mfuc6, 0.2 μg/ml Mfuc7, 0.3 μg/ml Thma. For reactions with 1.0 mM pNP-Fuc, with the exception of Mfuc2, the double concentration of enzyme was used. The release of pNP was monitored by withdrawing 50 μL samples at different time points (2, 4 and 8 min for 0.1 mM pNP-Fuc and 5, 10 and 20 min for 1.0 mM pNP-Fuc), stopping reactions by addition of 50 μL of 1 M sodium carbonate and measuring the absorbance at 405 nm. The pH vs hydrolase activity plots are shown in S1 Fig.

For a substrate saturation experiment, reactions were set up as described for the pH-dependence experiment (0.1 mM pNP-Fuc) except that the reactions were done with 0.1, 0.25, 0.5, 1.0, 2.5, and 5 mM pNP-Fuc, and in case of Mfuc2 0.3 μg/ml of enzyme was used. The optimum pH was used for each enzyme. Samples were withdrawn at 2.5, 5 and 10 min and reactions were done in triplicate. Non-linear regression analysis was performed with the use of GraphPad Prism (GraphPad Software, USA) and kinetic data for Mfuc6 were fitted using the allosteric model (Y = V_max_*X^h^/(K_prime_ + X^h^)) and the data for the remaining six enzymes with the substrate inhibition model (Y = V_max_*X/(K_m_ + X*(1+X/K_i_))) [23]. The substrate concentration vs. hydrolase activity plots are presented in S2 Fig.

For estimation of the temperature stability, the enzymes were incubated in 5 mM buffer at optimal pH at a concentration of 0.8 μg/ml Mfuc1, 0.4 μg/ml Mfuc2, 1.2 μg/ml Mfuc3, 0.9 μg/ml Mfuc4, 0.7 μg/ml Mfuc5, 3.9 μg/ml Mfuc6, 0.5 μg/ml Mfuc7, 0.9 μg/ml Thma wild type for 10, 20, 30 and 40 min at various temperatures in the range 30–100°C. Residual enzyme activity was measured in a continuous assay using 1 mM pNP-Fuc with 50 mM phosphate-citrate buffer (pH 7) and 10% of the enzyme concentration stated above and the release of pNP was monitored spectrophotometrically at 405 nm. Plots of heating time vs residual activity are presented in S3 Fig.

The substrate specificity of purified enzymes was analysed at 30°C using 1 mM of the following chromogenic substrates in 50 mM phosphate-citrate buffer (pH 7.0): pNP-β-D-Gal, pNP-β-D-Glc, pNP-β-D-Lac using the enzyme concentrations as described for the pH-dependence experiment. The release of pNP was followed spectrophotometrically at 405 nm in a continuous assay.

The hydrolysis of 2’-FL, 3-FL, and fucose-containing xyloglucan (27 mg fucose/g) was performed in duplicates in 96-deep well plates in 500 μl reactions at 30°C with 20 μg/ml enzyme (40 μg/ml for Mfuc7). For FLs, 1 mM substrate was used and reactions proceeded for 150 min. For xyloglucan, the substrate concentration was chosen to correspond to a final fucose content of 0.25 mM and reactions were carried out for 240 min. Post-reaction mixtures were filtered using AcroPrep Advance 96-Well Omega Filter and ultrafiltered using Ultrafilter (10 kDa MWCO) plates, respectively (Pall–USA, Port Washington). Diluted permeates were analysed by HPAEC-PAD. The results are presented as percent release of fucose compared to total fucose content in the substrate. Determination of hydrolytic activity on xyloglucan from *Arabidopsis thaliana* and *Sambucus nigra* was done using immune-glycan arrays. The extraction of cell wall components with sodium hydroxide, array printing and subsequent quantification using antibodies were performed essentially as previously reported [24]. The results are presented in percent where 0% corresponds to the signal from untreated samples whereas 100% corresponds to the complete α-L-fucosidase-mediated removal of the antibody fucosyl epitope detected by the antibody. A detailed protocol is given in S2 File.

### Trans-fucosylation assays

For initial evaluation of trans-fucosylation activity, non-buffered reactions were performed using 20 mM pNP-Fuc as the donor and 25 mM lactose as the acceptor at 30°C for 50 min. 500 μl reactions were started by the addition of enzymes to final concentration of 42 μg/ml Mfuc1, 12 μg/ml Mfuc2, 41 μg/ml Mfuc3, 34 μg/ml Mfuc4, 18 μg/ml Mfuc5, 65 μg/ml Mfuc6, 17 μg/ml Mfuc7 and 51 μg/ml Thma wild type. The reactions were stopped by removing the enzyme with Vivaspin 500 filters with a 5 kDa cut-off (Sartorius, Germany) at 4°C. The permeate was freeze-dried and analysed by nuclear magnetic resonance (NMR) spectroscopy.

Trans-fucosylation activity was evaluated in a time-course experiments using 25 mM pNP-Fuc as donor and 100 mM lactose as acceptor, at 30°C and pH optimum (as determined for hydrolysis of pNP-Fuc). Additionally, for Mfuc5, a reaction using a suspension of 33.7 g/L citrus peel xyloglucan (5.55 mM bound fucose), 140 mM lactose and 56 mM phosphate-citrate buffer (pH 7) was done at 30°C. The final enzyme concentrations were as for the transfucosylation experiment described above. The reactions were carried out in duplicates in 96-deep well microtiter plates. 30 μl samples were withdrawn at indicated time points and diluted 10-fold with ice-cold MilliQ water. The post-reaction mixtures were filtered using AcroPrep Advance 96-Well Omega Filter and ultrafiltered using Ultrafilter (10 kDa MWCO) plates, respectively (Pall, USA). The permeates were further diluted for determination of pNP content by spectrophotometry at 410 nm and HPAEC-PAD analysis.

### Nuclear magnetic resonance spectroscopy

Freeze-dried trans-fucosylation samples were dissolved in 500 μL D_2_O (99.9% D, Sigma-Aldrich) and NMR spectra were acquired in at 25°C on a Varian Unity Inova 500 MHz spectrometer equipped with a 5 mm probe or a Bruker Ascend 400 MHz spectrometer with a 5 mm Prodigy cryoprobe using standard pulse sequences. An external standard of 1,4-dioxane was used as chemical shift reference (δ_H_ 3.75 ppm and δ_C_ 67.4 ppm). Structure elucidation was achieved by full ^1^H and ^13^C NMR assignments using standard 1D and 2D homo- and heteronuclear NMR experiments. Linkage positions were determined by strong and unambiguous heteronuclear multiple bond correlations (HMBC) across the glycosidic bonds between the respective fucose residues, as well as from Fuc H1 to C1 of pNP. Nuclear Overhauser effect spectroscopy (NOESY) connectivities were used to confirm the structures [25,26]. Selected NMR spectra and NMR assignments for compounds annotated in this study are presented in the S3 File. Literature data for the (1–2)- and (1–4)-linked glycosides by Benešová et al. [27] were comparable to our observations, however with minor discrepancies on the chemical shift values, which may result from pH effects. The previously reported NMR data for the (1–3)-linked glycoside was done using pyridine-d5 [15] and the values are therefore not comparable to ours. The concentrations of observed trans-fucosylation products were estimated by comparison of characteristic non-overlapping ^1^H NMR signals and these of fucose in each sample (S3 File).

### High-performance anion exchange chromatography (HPAEC-PAD) and reverse phase chromatography (RP-HPLC)

A Dionex ICS-5000 system consisting of DP-5000 gradient pump, ED-5000 electrochemical (pulsed amperometric) detector coupled to an AS-AP autosampler (Dionex Corp., CA) was used for HPAEC-PAD analysis. The separation was accomplished using a CarboPac PA1 analytical column (4 mm × 250 mm) with CarboPac PA1 guard column (4 mm × 50 mm) (Dionex Corp.) at 30°C with a flow rate of 1 ml/min. Hydrolysis products of 2’-FL, 3-FL and high-molecular substrates were analysed with the following programme: 0–16 min at 75 mM NaOH, 17–21 min at 250 mM NaOH and regeneration 22–35 min at 75 mM NaOH. For analysis of products in transfucosylation reactions, a 40 min isocratic separation using 75 mM NaOH as eluent was performed.

To identify individual *para-*nitrophenyl α-L-fucopyranosyl-α-L-fucopyranoside products (Fuc-Fuc-pNP), transfucosylation reactions were performed as described for the time-course experiments with the use of Mfuc6 and Mfuc7. After enzyme removal, compounds in the 20 μl permeate samples were separated by RP-HPLC using an ODS-L Optimal column (250 mm x 4.6 mm) at 30°C and with a flow rate of 1 ml/min. A two-solvent system consisting of acetonitrile (A) and water (B) was used with a linear gradient from 3 to 30% A for 40 min and regeneration at 3% A for 15 min. The individual pNP-containing compounds were detected by UV spectrophotometry at 225 nm and collected into separate fractions. The procedure was repeated three times using each of the post-reaction mixtures. Fractions corresponding to individual peaks were pooled and freeze-dried. The fractions were analysed by ^1^H NMR and used as standards for HPAEC-PAD analysis as detailed above.

### Sequence analysis

To identify the closest homolog, protein sequences were used as query in BLAST_P_ for search against the non-redundant protein sequences database [22]. The protein sequences were also analysed using the NCBI Conserved Domain Database using the default settings [28]. To predict the CAZY glycosyl hydrolase family, the CAZYmes Analysis Toolkit was used with default settings [29]. A phylogenetic tree of the metagenome-derived proteins and select representatives of the GH 29 family was constructed. The following α-L-fucosidases were included in the analysis: AlfA (Uniprot B3W8U6), AlfB (Uniprot B3WB08) and AlfC (Uniprot B3WBB5) from *Lactobacillus casei*, BT_2192 (Uniprot Q8A5P6) and BT_2970 (Uniprot Q8A3I4) from *Bacteroides thetaiotaomicron*, TM306 (Uniprot Q9WYE2) from *T*. *maritima*, AfcB (Uniprot C5NS94) from *Bifidobacterium bifidum* JCM 1254, Blon_2336 (Uniprot B7GNN8), Blon_0248 (Uniprot B7GTT5) and Blon_0426 (Uniprot B7GN40) from *Bifidobacterium longum* ATCC15697, ALfuk1 from *Paenibacillus thiaminolyticus* (Uniprot E3PQQ9), BFO_2737 (Uniprot G8UMQ6) from *Tannerella forsythia* ATCC 43037, FucA1 (Uniprot Q8P6S7) from *Xanthomonas campestris* ATCC 33913, FUCA1 (Uniprot P48300) from *Canis lupus familiaris*, AlfA (Uniprot P10901) from *Dictyosetlium discoideum*, FCO1 from (Uniprot J9TMS7) *Fusarium oxysporum*, and FCO1 (Uniprot J9UN47) from *Fusarium graminearum* PH-1, α-1,3/1,4-L-fucosidase (Uniprot Q9Z4I9) from *Streptomyces* sp. 142, FucA1 (Uniprot Q97UG1) from *Sulfolobus solfataricus* P2, AtFuc1 (Uniprot Q8GW72) from *A*. *thaliana*, FucA1 (Uniprot P04066) and FucA2 (Uniprot Q7Z6V2) from *HHdHomo sapiens*, and FucA1 (Uniprot P17164) from *Rattus norvegicus*. A summary of published data on activities of the enzymes towards various substrates is presented in S2 Table. The neighbor-Joining method [30] was used to infer the phylogeny and evolutionary analyses were conducted in MEGA6 [31]. All positions containing gaps and missing data were eliminated. There were a total of 169 AA positions in the alignment. The percentage of replicate trees (>50%) in which the associated taxa clustered together in the bootstrap test (500 replicates) are shown next to the branches [32].

### Accession numbers

The complete nucleotide sequences of novel α-L-fucosidase-encoding genes are available in GenBank under the following accession numbers: KJ626336 (*mfuc1*), KJ626337 (*mfuc2*), KJ626338 (*mfuc3*), KJ626339 (*mfuc4*), KJ626340 (*mfuc5*), KJ626341 (*mfuc6*), and KJ626342 (*mfuc7*).

## Results

### Identification and sequence analysis of α-L-fucosidases by metagenome screening

In this study a soil metagenome library was screened for novel α-L-fucosidases. Of approximately 100,000 colonies screened using the chromogenic substrate 5-bromo-4-chloro-3-indolyl-α-L-fucopyranoside, α-L-fucosidase activity was detected in 40 clones. From these clones, fosmids were isolated and subjected to restriction analysis resulting in the identification of 16 non-redundant clones. Fosmid inserts of about 50 kb were digested with *Bam*HI or *Pst*I and the smaller restriction products were inserted in the vector pUC18 and screened on X-Fuc, yielding seven positive clones. The DNA was sequenced using Ion Torrent, allowing identification of seven α-L-fucosidase-encoding genes that were denoted *mfuc1*, *2*, *3*, *4*, *5*, *6* and *7*.

### Sequence analysis of metagenome-derived α-L-fucosidases

**Fig 1:**
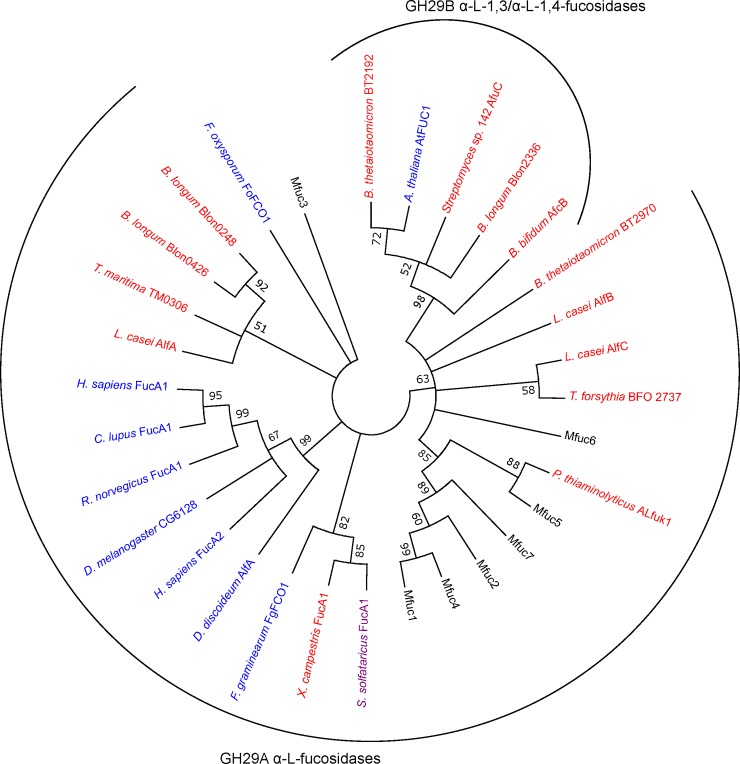
Phylogenetic tree of novel α-L-fucosidase sequences, Thma and characterised representatives of GH 29 family. Phylogeny was inferred using the neighbour-joining method. The percentage of replicate trees (>50%) in which the associated taxa clustered together in the bootstrap test (500 replicates) are shown next to the branches. Bacterial α-L-fucosidases are shown in red, eukaryotic in blue and the archeal α-L-fucosidase in purple. Accession numbers for the enzymes included in the analysis are provided in the materials and methods section.

Firstly, we performed a BLAST_P_ analysis. This demonstrated that all enzymes, with exception of Mfuc3, showed sequence identity with known α-L-fucosidases in the range of 65–76% (Table 1). Mfuc3 shared only 41% identity with the closest homolog, a β-galactosidase trimerisation domain-containing protein. Most of the α-L-fucosidases had a theoretical molecular weight between 47.3 and 52.2 kDa, while Mfuc3 was larger with a molecular weight of 75.6 kDa. The Mfuc sequences were analysed using the NCBI Conserved Domain software [28] and all except Mfuc3 were found to contain an α-L-fucosidase domain (pfam01120). Significant hits for Mfuc3 were GHL6 (pfam14871), a family of hypothetical glycosyl hydrolases, and to A4 beta-galactosidase middle domain. Using the sequence-based annotation tool in the CAZYmes Analysis Toolkit [29], all identified enzymes, were classified in GH 29. To make an assessment of the sequence diversity between the metagenome-derived α-L-fucosidases and the most established representatives of GH 29 family [33], we constructed a phylogram (Fig 1). A few well-defined sequence clusters could be observed. The first one constituted enzymes previously characterized as unable to cleave pNP-Fuc and possessing capability to cleave α-1,3/α-1,4 linkages. This cluster consists of members of GH 29B. Most eukaryotic sequences clustered together, all of them having wide substrate specificity, cleaving a number of α-1,2/ α-1,3 and α-1,4 linkages. As can be observed, Blon02448, and Blon0426 from *B*. *longum*,and AlfA from *L*. casei with no capability to cleave α-1,2 and α-1,3 linkages but hydrolyzing pNP-Fuc also exhibit close phylogenetic relationship. They also cluster with Thma analysed in this study. As expected from the relatively low homology to known enzymes in the BLAST analysis, the α-L-fucosidases did not cluster with the established members of the fucosidase family. Instead Mfuc1, 2, 4, 5 and 7 formed a new clade, together with *P*. *thiaminolyticus* aLfuk1, that previously was shown to catalyze synthesis of various fucosylated glycosides using pNP-Fuc as donor [26]. Mfuc3 and Mfuc6 did not cluster with any of the enzymes included in the analysis. It has been shown that the catalytic nucleophile in GH 29 enzymes is a conserved aspartate residue [10]. This residue was also identified in all of the α-L-fucosidases reported here by multiple sequence alignment (S4 Fig).

**Table 1:** BLASTp analysis of metagenome-derived protein sequences.

Gene	Identity (similarity, %)	Best hit (accession no.)	Organism
*mfuc1*	67 (81)	α-L-fucosidase (YP_001278245)	*Roseiflexus* sp. RS-1
*mfuc2*	73 (83)	α-L-fucosidase (YP_003323631.1)	*Thermobaculum terrenum*
*mfuc3*	41 (57)	β-galactosidase trimerisation domain-containing protein (YP_004464268.1)	*Mahella australiensis*
*mfuc4*	70 (81)	α-L-fucosidase (YP_001278245)	*Roseiflexus* sp. RS-1
*mfuc5*	74 (85)	Putative α-L-fucosidase (YP_004173881.1)	*Anaerolinea thermophila*
*mfuc6*	65 (81)	α-L-fucosidase (YP_007147579.1)	*Cylindrospermum stagnale*
*mfuc7*	76 (86)	α-L-fucosidase (WP_009252575.1)	*Lachnospiraceae bacterium*

The identity and similarity in % to the closest homolog in the NCBI database. Organism and accession numbers are given.

### Production of recombinant α-L-fucosidases in *E*. *coli*

In order to evaluate the hydrolysis and trans-fucosylation properties of the seven metagenome-derived α-L-fucosidases as well as that of *T*. *maritima* (Thma), we produced them in *E*. *coli*. The genes were inserted in the pETM10 vector and were expressed in *E*. *coli* BL21(DE3) and C41(DE3). The highest yields of recombinant proteins were obtained for Mfuc1, 2 and 4 when expressed in *E*. *coli* BL21(DE3) and for Mfuc3, 5, 6 and 7 when expressed in *E*. *coli* C41(DE3). SDS-PAGE of purified 6xHis-tagged α-L-fucosidases verified the expected size of enzymes (S5 Fig).

### Thermostability, pH optimum, and apparent kinetics of pNP-Fuc hydrolysis

As expected, all the enzymes were able to hydrolyse the frequently used fucosidase test substrate pNP-Fuc. As Mfuc3 appeared to be related to β-galactosidases, we tested all enzymes on an array of glycosyl hydrolase substrates (pNP-β-D-Gal, pNP-β-D-Glc, pNP-β-D-Lac), but none of the enzymes were able to hydrolyse these substrates. An assessment of physicochemical properties of the enzymes was done using pNP-Fuc as substrate. Most enzymes were active in a relatively broad pH range and exhibited maximum activity at pH 6–7, whereas the optimum for Mfuc6 was pH 9 (Table 2). Next, we tested the thermostability of the enzymes, and while some differences in stability were observed, all metagenome-derived enzymes had a half-life of less than 5 min at 60°C. In contrast, Thma, derived from the hyperthermophilic bacterium *T*. *maritima*, had a half-life of about 48 min at 100°C.

**Table 2:** Properties of metagenome-derived α-L-fucosidases and Thma.

	Mfuc1	Mfuc2	Mfuc3	Mfuc4	Mfuc5	Mfuc6	Mfuc7	Thma
**pH opt**	7	7	6	7	7	9	6	7 ^**1**^
**pH interval**	6–9	6–10	6–8	6–9	5–8	6–10	6–7	4–9
**Thermal half life**								
30°C	> 2 h	> 2 h	> 2 h	> 2 h	> 2 h	> 2 h	~98 min	> 2 h
40°C	> 2 h	> 2 h	> 2 h	> 2 h	> 2 h	~56 min	~28 min	> 2 h
50°C	> 2 h	~9 min	~67 min	> 2 h	< 2 min	~14 min	~8 min	> 2 h
60°C	< 2 min	< 2 min	~4 min	< 2 min	< 2 min	< 2 min	< 2 min	> 2 h
**Kinetic parameters** ^**2**^								
V_max_	1.6±0.0	2.3 ±0.1	1.6 ±0.1	0.77±0.02	1.9±0.04	0.56±0.05	2.2±0.16	0.67±0.02
K_m_ (K_prime_)	0.11±0.01	0.14±0.01	0.31±0.03	0.071±0.01	0.13±0.01	0.40±0.05	0.28±0.05	0.06±0.01
K_i_ (h)	7.1±0.6	7.1 ±0.9	2.6 ±0.3	30 ±6.8	12±1.2	0.74±0.16	12 ±3.4	22 ±4.9

^1^ pH optimum was measured with 0.1 and 1 mM substrate; for Thma two optima (pH 4 and 7) were observed with 1 mM substrate.

^2^ Kinetic parameters were determined using pNP-Fuc as substrate at 30°C at the optimal pH.

Plots of pH vs. hydrolase activity, substrate concentration vs. hydrolase activity, and heating time vs. residual activity for the enzymes are presented in S1 Fig, S2 Fig and S3 Fig.

Using pNP-Fuc as a substrate, the pH optimum, the pH interval (where the enzyme displayed more than 20% of maximal enzyme activity) and thermal half-lives at relevant temperatures were determined. The kinetic parameters for hydrolysis of pNP-Fuc at 30°C were determined. Most enzymes displayed substrate inhibition and estimated V_max_, K_m_ and K_i_ are reported. In the case of Mfuc6, the kinetic data were fitted using the allosteric model (Y = V_max_*X^h^/(K_prime_ + X^h^) and V_max_, K_prime_ and h is reported.

Several characterized α-L-fucosidases have been shown to be substrate-inhibited when pNP-Fuc is used as a substrate [11,27]. We therefore determined the kinetic constants and found that the K_m_ values for the eight enzymes ranged from 0.07 mM to 0.31 mM (Table 2). All enzymes, except Mfuc6, exhibited some level of substrate inhibition with pNP-Fuc as substrate, with K_i_ values ranging between 2.6 and 30.1 mM. For Mfuc6, the substrate-saturation data was best modeled using an allosteric sigmoidal model, and the Hill (H) coefficient of 0.40 indicated a negative cooperativity in substrate binding (S2 Fig).

### Substrate specificity

Having characterized the enzymes for various physicochemical properties, we then considered the substrate specificity of the enzymes. To test the regioselectivity of the enzymes, we measured their hydrolase activity on the two fucosylated HMOs, 2’-FL (α-1,2-linked to galactose) and 3-FL (α-1,3-linked to glucose). While Mfuc3 and 6 were unable to hydrolyse either of the substrates, the remaining six enzymes all showed a preference for the α-1,2-linkage (Table 3). In contrast, only Mfuc5 displayed relatively high activity with 3-FL, while a low activity was observed for Mfuc1 and Mfuc7.

**Table 3:** Determination of regiospecificity and substrate specificity for hydrolysis of natural oligosaccharides and glycans.

	Mfuc1	Mfuc2	Mfuc3	Mfuc4	Mfuc5	Mfuc6	Mfuc7	Thma
2’-FL	76 ±1%	88±1%	2 ±1%	47±1%	87±1%	2 ±2%	94±1%	71±1%
3-FL	11 ±2%	4±2%	0 ±2%	3 ±2%	34 ±3%	0 ±2%	12 ±2%	1 ±2%
Citrus XG	4%	1%	1%	3%	39%	4%	3%	0%
*Arabidopsis* XG	100%	84%	7%	78%	100%	7%	43%	13%
*Sambucus* XG	100%	85%	7%	91%	100%	5%	44%	9%

The reactions were performed using 1 mM of substrate at 30°C for 150 min and the fraction of fucose released from the substrates under the reaction conditions employed is given in %. For hydrolysis of 2’-FL, 3-FL and citrus xyloglucan, the fucose content was determined by HPAEC-PAD. The hydrolysis of fucose from *Arabidopsis thaliana* and *Sambucus nigra* xyloglucan was measured using immuno-glycan arrays (S2 File), and 0% corresponds to the signal from untreated samples whereas 100% corresponds to the complete α-L-fucosidase-mediated removal of the antibody fucosyl epitope detected by the antibody.

Having established the regioselectivity of the enzymes, we wanted to test whether the enzymes were able to act on the natural polymer xyloglucan. In xyloglucan, fucose is bound to galactose in an α-1,2-linkage [14]. When tested on xyloglucan extracted from citrus peel, only Mfuc5 exhibited a substantial activity, releasing ~39% of the total fucose content (Table 3). When tested on xyloglucan derived from the two flowering plants *A*. *thaliana* and *S*. *nigra*, five enzymes, Mfuc1, 2, 4, 5 and 7, were able to release fucose, indicating source-dependent differences in the xyloglucan structure.

### Trans-fucosidase activity of metagenome α-L-fucosidases

**Fig 2:**
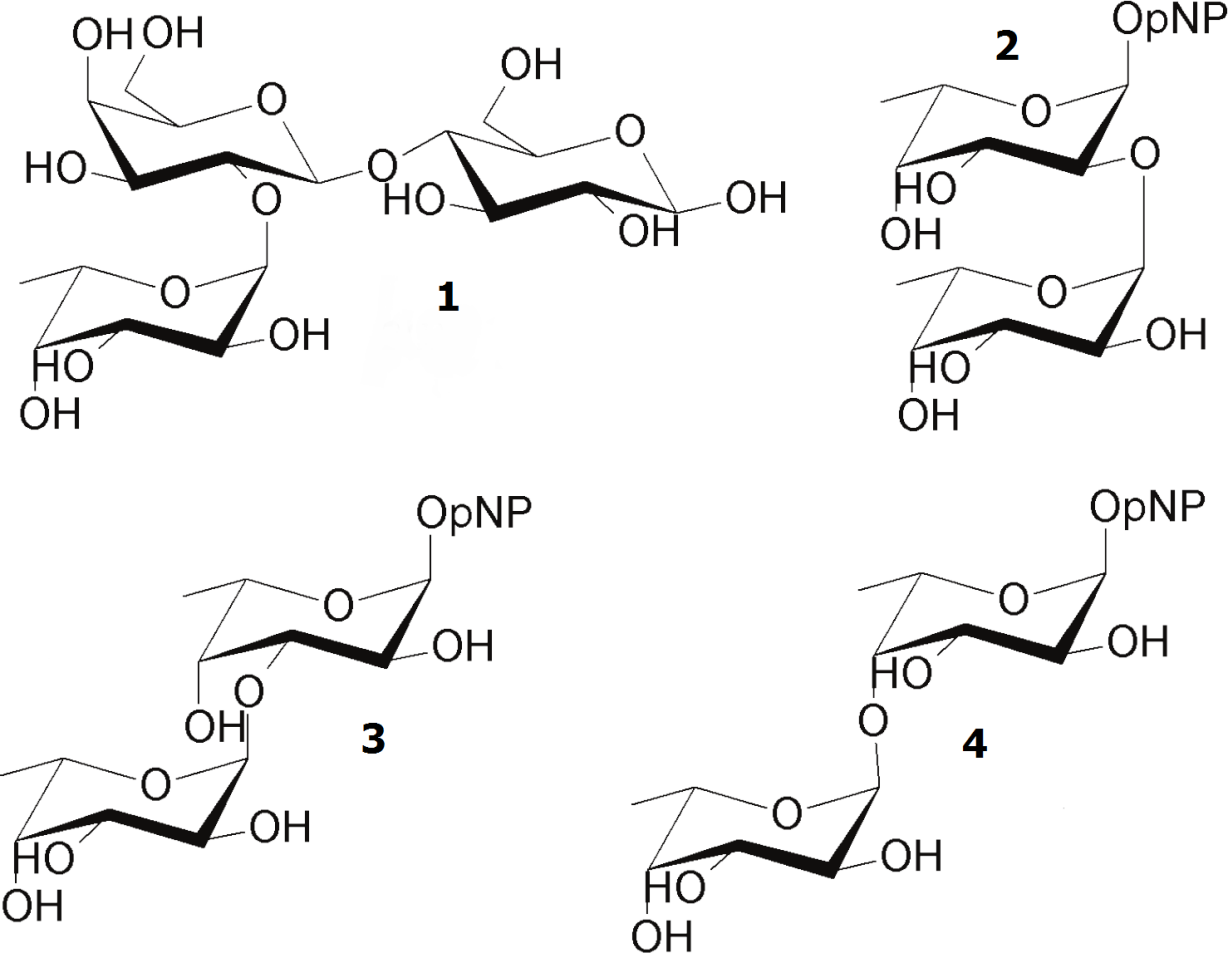
Structure of reaction products from enzymatic trans-fucosylation. In addition to an undetermined FL, **1:** the HMO 2’-FL and the self-condensation products **2**: Fuc*p*-(1–2)-Fuc*p*-pNP, **3**: Fuc*p*-(1–3)-Fuc*p*-pNP, **4**: Fuc*p*-(1–4)-Fuc*p*-pNP were synthesised.

Having analysed the substrate specificity, we finally tested the enzymes for trans-fucosylation activity. For an initial evaluation of trans-fucosidase activity, reactions containing 20 mM pNP-Fuc as donor and 25 mM lactose as acceptor were run for a fixed time and analysed by HPAEC-PAD and NMR. HPAEC-PAD revealed that different FLs including the genuine HMO compounds 2’-FL were produced. Thma catalysed synthesis of 0.19 mM 2’-FL, while Mfuc5 synthesised FL in a yield of 0.24 mM, in both cases corresponding to yields of ~1% yield (Table 4). The product of Mfuc5 was analysed by both NMR and liquid chromatography-mass spectrometry. NMR did not yield conclusive results due to interference from the high excess of lactose. By liquid chromatography-mass spectrometry we confirmed that it was FL, and could rule out that it was 2’-FL or 3-FL (the only FLs for which standards are commercially available). The remaining six enzymes produced none, or lower amounts of the two FLs. Three different self-condensation products were also identified by NMR, corresponding to *p*-nitrophenyl α-L-fucopyranosyl-(1–2)-α-L-fucopyranoside as well as the (1–3)- and (1–4)-linked Fuc-Fuc-pNP glycosides. An overview of the trans-fucosylation reaction products is presented in Fig 2. Fuc-(1–2)-Fuc-pNP was the predominant product for most enzymes, but Mfuc7 produced preferentially Fuc-(1–3)-Fuc-pNP. In the reaction catalysed by Mfuc6, 5.7 mM Fuc-(1–2)-Fuc-pNP was produced corresponding to a yield of 34% based on the donor.

**Table 4:** Fucosylated products from trans-fucosylation reactions.

Enzyme	Concentration (mM)						
	pNP-Fuc	Fucose	2’-FL	FL^1^	Fuc-Fuc-pNP		
					α-1,2	α-1,3	α-1,4
Mfuc1	0.12	20.3	0.00	0.05	0.09	0.00	0.00
Mfuc2	0.08	17.9	0.07	0.08	0.61	0.00	0.00
Mfuc3	0.00	18.3	0.00	0.02	0.13	0.03	0.00
Mfuc4	0.44	18.3	0.00	0.04	0.18	0.00	0.00
Mfuc5	0.45	12.9	0.03	0.24	0.49	0.20	0.04
Mfuc6	1.17	7.3	0.00	0.01	5.67	0.32	0.75
Mfuc7	4.58	12.3	0.00	0.02	1.17	1.99	0.00
Thma	1.02	13.9	0.19	0.00	3.40	0.06	0.06

^1^ FL is fucosyllactose other than 2’-FL and 3-FL.

The reactions were performed at 30°C for 50 min using 20 mM pNP-Fuc as donor and 25 mM lactose as acceptor. Concentrations of substrates and products in trans-fucosylation reactions was determined using HPAEC-PAD and NMR. Concentrations of pNP-Fuc and and Fuc- Fuc-pNP was estimated by comparison of characteristic non-over-overlapping ^1^H NMR signals and that of L-fucose.

**Fig 3:**
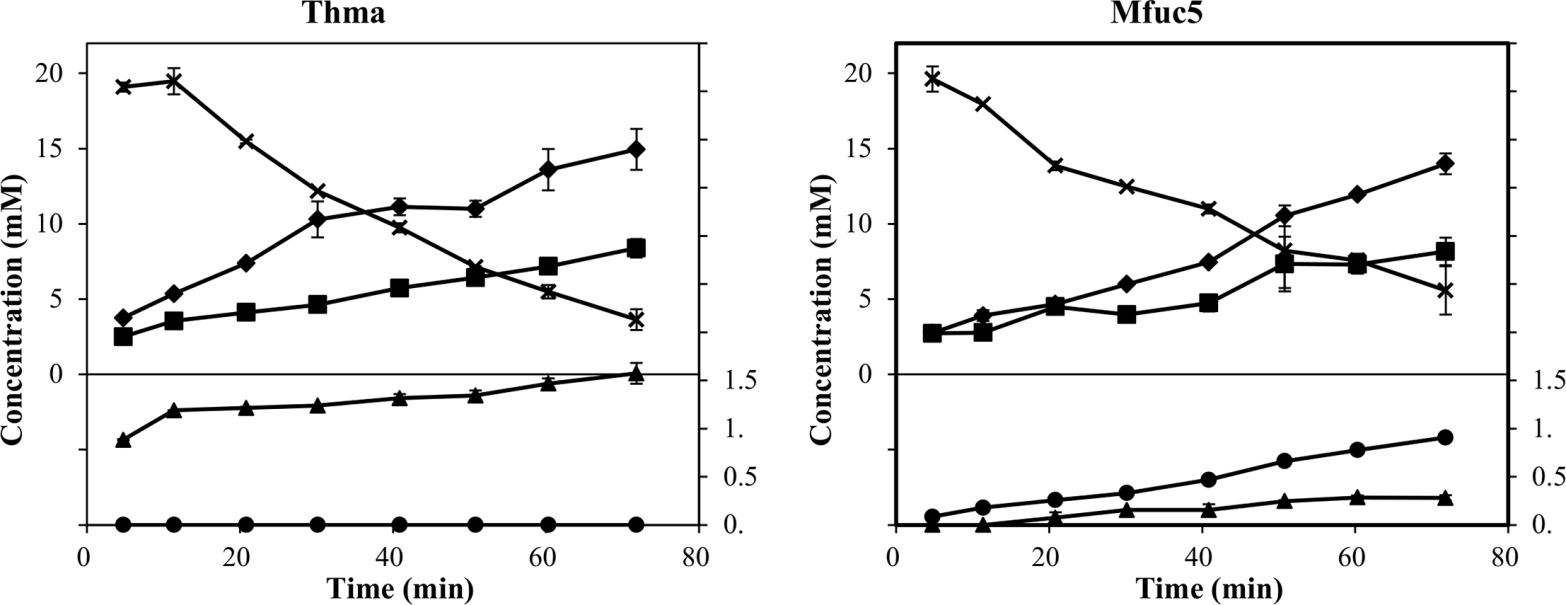
Time study of trans-fucosylation catalysed by Thma and Mfuc5. Reactions were done using 25 mM pNP-Fuc as donor and 100 mM lactose as acceptor at 30°C and optimal pH. The concentrations were determined by HPAEC-PAD and for pNP by spectrophotometry. L-fucose (■), pNP (⧫), pNP-Fuc (X), 2’-FL (▲), and 3-FL (●).

In trans-glycosylation reactions using glycosyl hydrolases, careful control of reaction time is often important due the inherent hydrolase activity that affects yield by degradation of both donor and product molecules [8]. It has also been demonstrated for other hydrolases that a high concentration of acceptor can improve transglycosylation yields [12,34]. We therefore set up reactions with a higher acceptor:donor ratio (100 mM lactose as acceptor and 25 mM pNP-Fuc as donor) and followed the reactions in time course-experiments. The transient maximum yield of FL present in the reaction catalysed by Mfuc5 was 0.9 mM (~3.6% yield) whereas 1.6 mM (~6.4% yield) of 2’-FL was produced in the reaction with Thma (Fig 3). The other enzymes were also analysed, but all produced less than 0.3 mM of FL (S6 Fig). Throughout the course of the reaction catalysed by Mfuc6, fucose concentration remained very low compared to concentration of pNP released, indicating predominant synthesis of self-condensation products.

**Fig 4:**
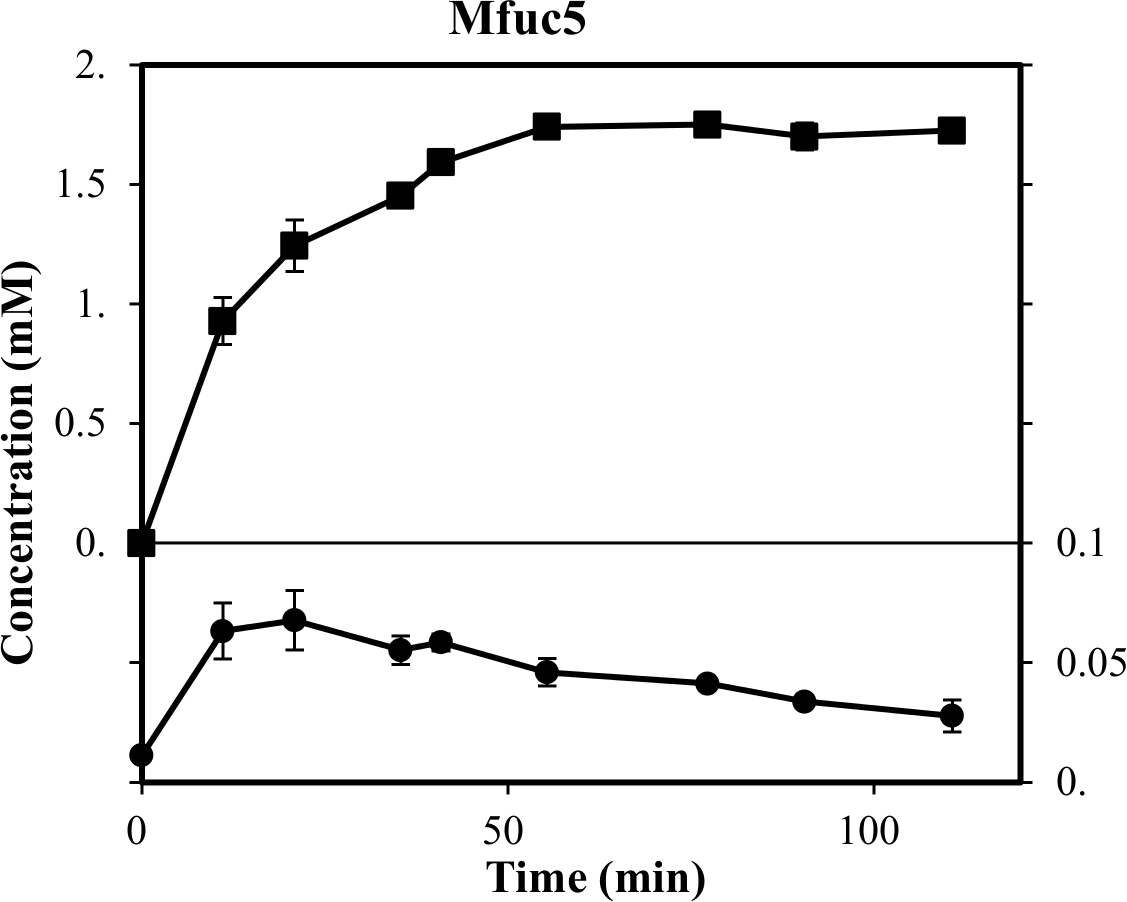
Time study of trans-fucosylation with xyloglucan as fucosyl donor catalyzed by Mfuc5. Reaction was done using 5.5 mM xyloglucan-bound fucose as donor and 140 mM lactose as acceptor at 30°C and pH 7. Concentration of L-fucose (■), and FL (●) was determined by HPAEC-PAD.

As Mfuc5 was capable of both releasing fucose from citrus xyloglucan and catalysing transfucosylation, we tested whether this natural polymer could be used as fucosyl donor for synthesis of FL. In the reaction we used a suspension of xyloglucan containing ~5.5 mM bound fucose and 140 mM lactose. A transient maximum FL concentration of 0.07 mM was observed in this experiment and a total of 1.8 mM fucose was released (Fig 4). This corresponded to a yield of ~1.3% with respect to total fucose and ~3.9% with respect to the enzymatically accessible fucose, which was comparable to the yield obtained using pNP-Fuc as fucosyl-donor.

## Discussion

The primary aim of this study was to identify enzymes capable of hydrolysing fucose from plant-derived polymers and having trans-fucosylation activity for the production of fucosylated HMOs. Although a choice of methodology imposes a bias, functional genomics provides a relatively simple way to sample a larger diversity in the protein sequence space [18]. Using this approach, we identified seven α-L-fucosidases, all with relatively low homology (41–76% identity) to previously known enzymes. α-L-fucosidases are currently classified in the two glycoside hydrolase families GH 29 and GH 95. In this study, only GH 29 α-L-fucosidases were identified. α-L-fucosidases of GH 95 display no activity on pNP-Fuc and consequently the use of an artificial test substrate in the metagenome screen might have imposed a bias against this class of enzymes [35]. So far no GH 95 family fucosidases have been reported to catalyse trans-glycosylation. The fact that the natural substrate xyloglucan as well as the target product (FL) have the same stereochemistry of the anomeric bond would make these inverting enzymes less obvious candidates for trans-glycosylation. Recently, the GH 29 family was divided into two groups based on substrate specificity and phylogenetic clustering [33]. Subfamily A contains fucosidases that have relatively relaxed substrate specificities, whereas fucosidases from subfamily B are more specific for α-1,3 or α-1,4 linkages [33]. According to this classification, the α-L-fucosidases identified in this study belong to subfamily GH 29A. In general, there was some correlation between enzyme function and phylogenetic relationship within GH29 family. As observed in the phylogenetic tree with some of the best characterised representatives of this family (Fig 1), Mfuc1, 2, 4, 5, and 7 formed a new clade together with ALfuk1 from *P*. *thiaminolyticus* indicating a relatively close evolutionary relationship. ALfuk1 was previously shown to catalyse trans-glycosylation using pNP-Fuc as substrate [26]. In contrast, Thma clustered with enzymes previously shown not to act on α-1,2 and α-1,3 linkages whereas Thma in this study was shown to act on 2’-FL. Mfuc3 and Mfuc6 were more distantly related to the other α-L-fucosidases, and both exhibited low/no activity on the substrates tested with exception of pNP-Fuc. It therefore remains to be shown whether these enzymes are truly α-L-fucosidases, and if so, what natural substrates they target. The physicochemical properties of the enzymes were similar to those reported previously, that is, they were mesophilic enzymes with pH optimum in the range of pH 6–7 and exhibited substrate inhibition [11,27]. An interesting observation was that Mfuc6 had a pH optimum of 9 which, according to the Brenda Enzyme database [36] currently containing 86 records of pH optimum for α-L-fucosidases, is the highest one reported to date.

Due to the functional properties of fucosylated compounds, their specific synthesis is receiving increasing interest. Enzymatically, this can be done by employing trans-fucosidase activity of certain α-L-fucosidases. For industrial production of fucosylated oligosaccharides such as HMOs, it is relevant to identify inexpensive donor substrates. We consider the fucose-containing, natural polymer xyloglucan to be an interesting fucosyl-donor candidate. In xyloglucan, fucose is α-1,2-linked to galactose and thus resembles the structure observed in 2’-FL [14]. The ability of some α-L-fucosidases to hydrolyse xyloglucan has been demonstrated previously [37]. The metagenome-derived α-L-fucosidases and Thma were tested for activity on three xyloglucan substrates, and Mfuc1, 2, 4, 5, and 7 showed activity on xyloglucan.

For the evaluation of trans-fucosylation we used the principal fucosylated HMOs, 2’-FL and 3-FL, as model compounds. Thma was previously shown to catalyse trans-fucosylation but only with the artificial pNP-gal as acceptor [15]. We therefore included it in our analysis to test its trans-fucosylation ability with the more relevant acceptor substrate lactose. In an initial experiment, we identified Thma and Mfuc5 as the best catalysts for synthesis of fucosyllactose (Table 4). Interestingly, we also observed that the majority of the enzymes catalysed formation of self-condensation products where pNP-Fuc functions as both donor and acceptor to yield pNP-Fuc-Fuc with various linkages. Mfuc6 was unable to hydrolyse any of the substrates tested in this study and did not produce FL, but in contrast generated the highest yield of self-condensation products (33.7%). For enzymatic synthesis of fucosylated glycans, the self-condensation reaction however constitutes an undesirable side-product. It has been observed for most glycosidases, including α-L-fucosidases, that the +2 acceptor binding site exhibits a preference for pNP or an aromatic aglycon part likely related to a high density of aromatic residues [15]. This suggests that enzyme engineering of the acceptor binding site potentially could modulate the acceptor substrate specificity to favor fucosylation of lactose.

When the two enzymes Thma and Mfuc5 were analysed in a time-course experiment using 25 mM pNP-Fuc as donor and 100 mM lactose as acceptor, the transient maximum concentrations were 1.6 mM 2’-FL and 0.9 mM FL, corresponding to ~6.4% and ~3.6% yields respectively (Fig 3). α-L-fucosidases from various sources have been reported to catalyse trans-fucosylation reactions, using artificial donor substrates, with higher yields than reported here. Using *Alcaligenes* sp. α-L-fucosidase and lactose as acceptor, a yield of 34% of the non-natural 3’-FL was obtained [12]. With the monosaccharide N-acetylglucosamine as donor, *L*. *casei* α-L-fucosidases AlfB and AlfC synthesized the disaccharides fucosyl-α-1,3-N-acetylglucosamine and fucosyl-α-1,6-N-acetylglucosamine in yields of 23 and 56% [38]. It would be interesting to assess the performance of AlfB and AlfC on larger structures such as e.g. Lacto-N-tetraose.

The attractiveness of enzymatic synthesis of HMOs and other fucosylated compounds for use as food ingredients is intimately linked to the production cost. Therefore the application of inexpensive, natural fucosyl donors is desirable. Towards this goal, we have here, for the first time, demonstrated the use of a natural polymer, xyloglucan, as a donor for the synthesis of FL. The yield, compared to total fucose released, was ~3.9% which is similar to the yield obtained using pNP-Fuc as donor. Optimisation of the process was not considered in this study, but a number of strategies such as addition of organic solvents, optimisation of acceptor:donor ratio, enzyme load and ionic strength have previously led to significant improvement of yields in trans glycosylation reactions [8]. The yields can also be improved by increasing trans-glycosylation and/or reduce hydrolase activity by enzyme engineering as exemplified for *T*. *maritima* α-L-fucosidase [15]. Further, due to low solubility of the citrus-peel xyloglucan, further discovery of high-fucose containing plant cell wall xyloglucans and possibly substrate pre-treatment will be necessary to increase the industrial applicability of this enzymatic process.

In conclusion, our results further substantiate that functional mining of metagenomes can lead to the successful discovery of diverse glycoside hydrolases. Using recombinant α-L-fucosidases we were able to synthesize FLs including the fucosylated HMO 2’-FL. We also demonstrated for the first time, the potential of using the natural, fucose-containing polymer xyloglucan from citrus peel as a substrate for trans-fucosylation.

## Supporting Information

S1 Fig pH vs Hydrolase activity using pNP-Fuc as substrate. (PDF)

S2 Fig Substrate concentration vs. hydrolase activity plots using pNP-Fuc as substrate. (PDF)

S3 Fig Heat inactivation of α-L-fucosidases. (PDF)

S4 Fig Multiple sequence alignment of α-L-fucosidases reported in this study. (PDF)

S5 Fig SDS-PAGE of recombinant Mfuc1-7 and Thma. (PDF)

S6 Fig Time study of trans-fucosylation catalysed by metagenome-derived α-L-fucosidases. (PDF)

S1 File Sequence of codon-optimised Thma-encoding gene. (PDF)

S2 File Immuno-glycan microarray analysis of fucosylated xyloglucan cleavage. (PDF)

S3 File Selected NMR spectra, NMR chemical shifts and comparison of ^1^H NMR integrals. (PDF)

S1 Table List of primers used for amplification of α-L-fucosidase-encoding genes. (PDF)

S2 Table Substrate specificity of characterized GH29 fucosidases. (PDF)
