# Supplementary material for: Novel α-L-Fucosidases from a Soil Metagenome for Production of Fucosylated Human Milk Oligosaccharides
Source: PLoS One. 2016 Jan 22;11(1):e0147438. doi: 10.1371/journal.pone.0147438 (PMC4723247; doi:10.1371/journal.pone.0147438)
Supplement: S1 Fig — (PDF) [file pone.0147438.s001.pdf]

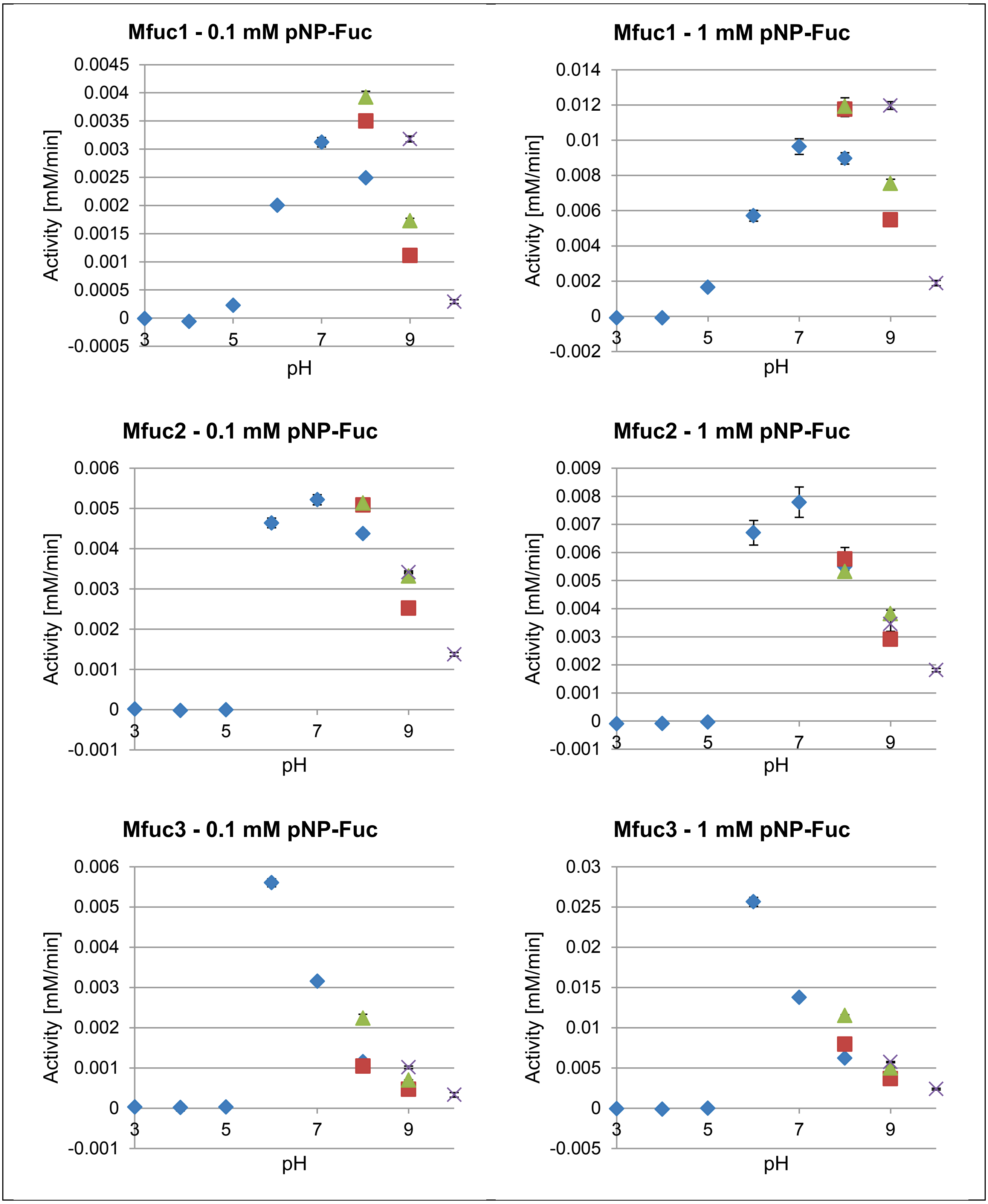
Mfuc1 - 0.1 mM pNP-Fuc
Activity [mM/min]
0.0045
0.004
0.0035
0.003
0.0025
0.002
0.0015
0.001
0.0005
0
-0.0005
3
5
7
9
pH
Mfuc1 - 1 mM pNP-Fuc
Activity [mM/min]
0.014
0.012
0.01
0.008
0.006
0.004
0.002
0
-0.002
3
5
7
9
pH
Mfuc2 - 0.1 mM pNP-Fuc
Activity [mM/min]
0.006
0.005
0.004
0.003
0.002
0.001
0
-0.001
3
5
7
9
pH
Mfuc2 - 1 mM pNP-Fuc
Activity [mM/min]
0.009
0.008
0.007
0.006
0.005
0.004
0.003
0.002
0.001
0
-0.001
3
5
7
9
pH
Mfuc3 - 0.1 mM pNP-Fuc
Activity [mM/min]
0.006
0.005
0.004
0.003
0.002
0.001
0
-0.001
3
5
7
9
pH
Mfuc3 - 1 mM pNP-Fuc
Activity [mM/min]
0.03
0.025
0.02
0.015
0.01
0.005
0
-0.005
3
5
7
9
pH

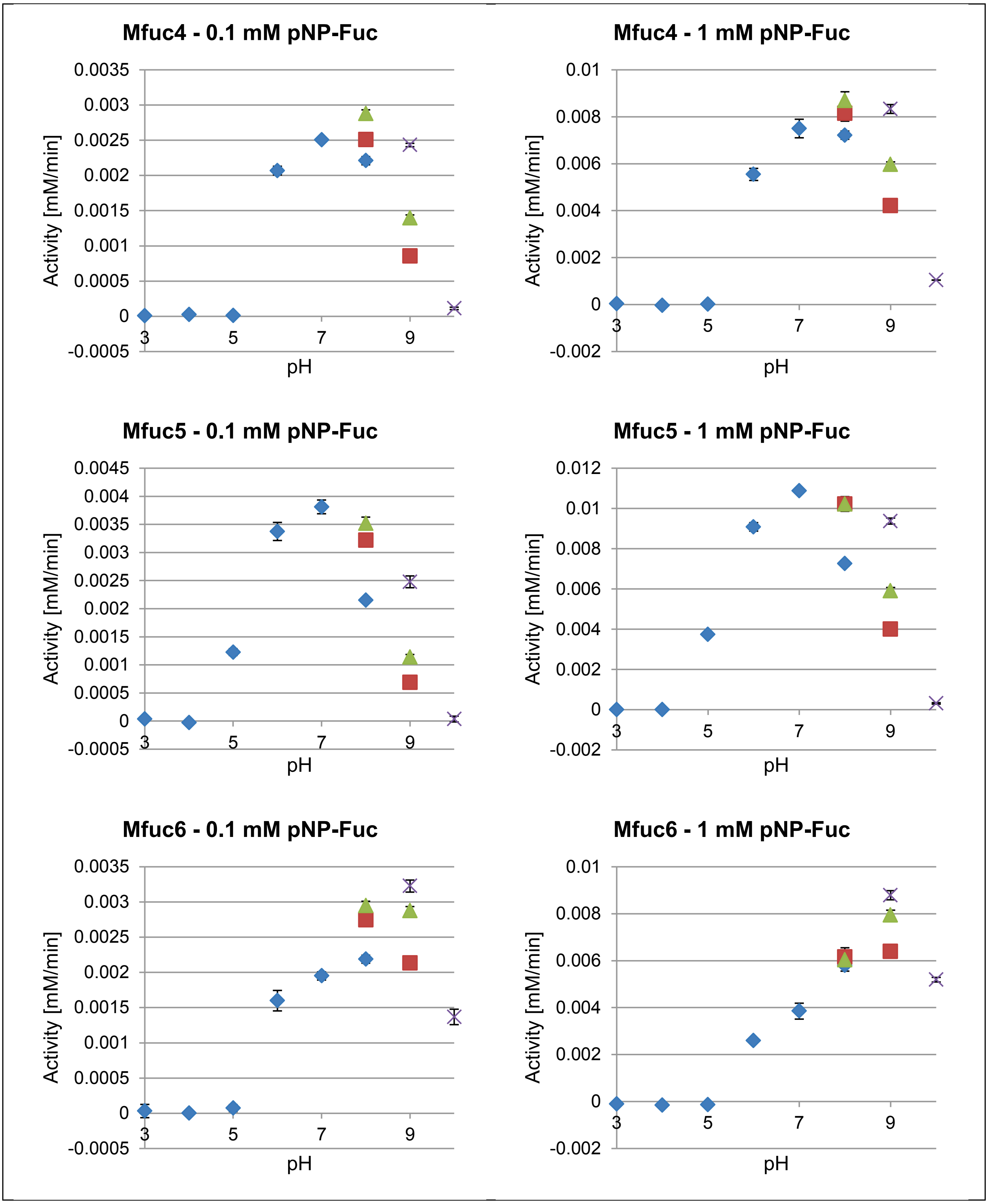
Mfuc4 - 0.1 mM pNP-Fuc
Activity [mM/min]
0.0035
0.003
0.0025
0.002
0.0015
0.001
0.0005
0
-0.0005
3
5
7
9
pH
Mfuc4 - 1 mM pNP-Fuc
Activity [mM/min]
0.01
0.008
0.006
0.004
0.002
0
-0.002
3
5
7
9
pH
Mfuc5 - 0.1 mM pNP-Fuc
Activity [mM/min]
0.0045
0.004
0.0035
0.003
0.0025
0.002
0.0015
0.001
0.0005
0
-0.0005
3
5
7
9
pH
Mfuc5 - 1 mM pNP-Fuc
Activity [mM/min]
0.012
0.01
0.008
0.006
0.004
0.002
0
-0.002
3
5
7
9
pH
Mfuc6 - 0.1 mM pNP-Fuc
Activity [mM/min]
0.0035
0.003
0.0025
0.002
0.0015
0.001
0.0005
0
-0.0005
3
5
7
9
pH
Mfuc6 - 1 mM pNP-Fuc
Activity [mM/min]
0.01
0.008
0.006
0.004
0.002
0
-0.002
3
5
7
9
pH

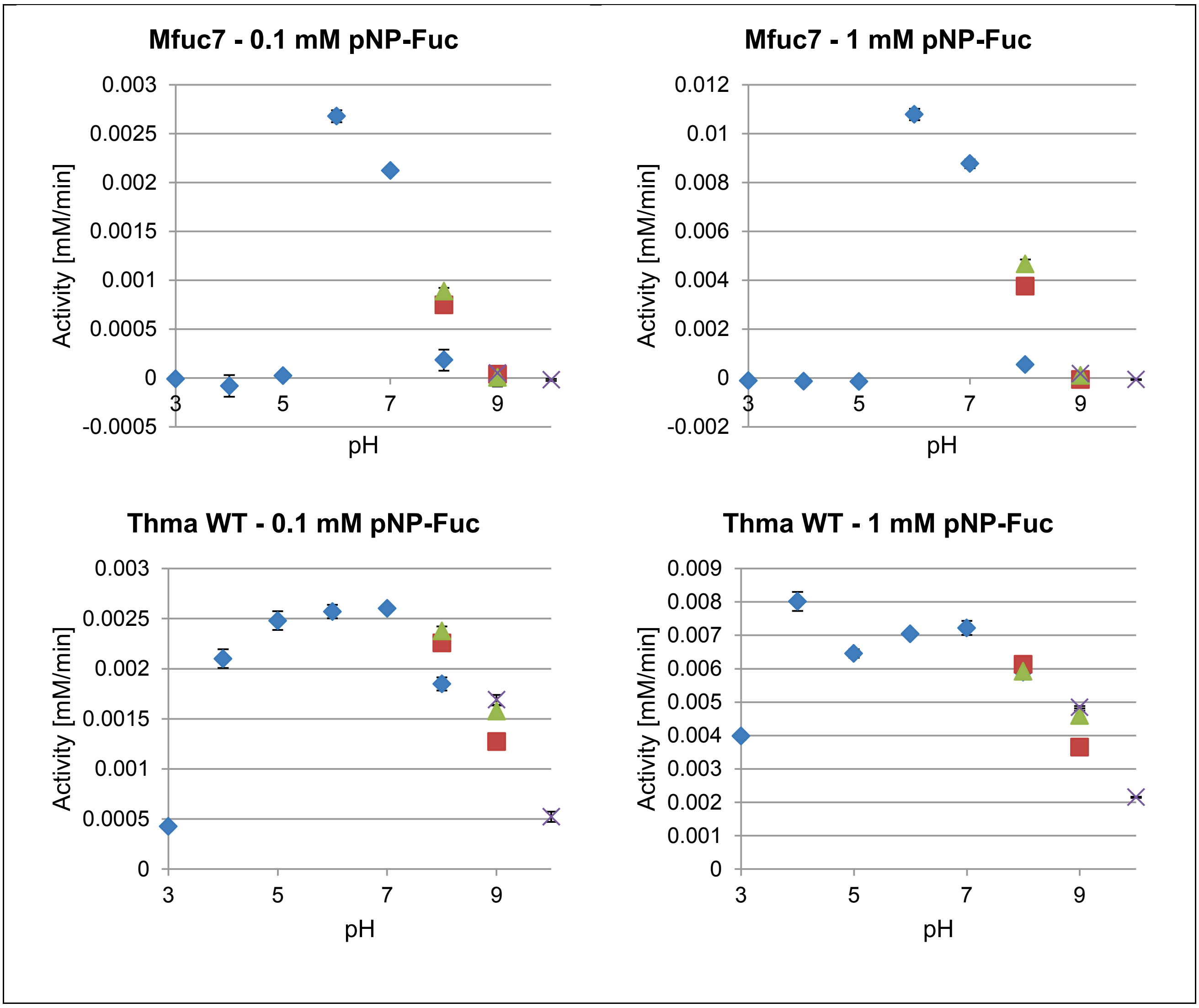

S1 Fig. pH vs Hydrolase activity using pNP-Fuc as substrate. The activity of the enzymes was tested at indicated pH and initial reaction rates were measured. The buffers used were 50 mM of phosphate-citrate (pH 3-8; blue diamonds), tricine (pH 8-9; red squares); glycylglycine (pH 8-9; green triangles), and glycine-NaOH (pH 9-10; purple crosses). Enzyme concentrations used in assays with 0.1 mM substrate were as follows: 0.2 µg/mL Mfuc1, 0.2 µg/mL Mfuc2, 0.6 µg/mL Mfuc3, 0.3 µg/mL Mfuc4, 0.2 µg/mL Mfuc5, 1.0 µg/mL Mfuc6, 0.2 µg/mL Mfuc7, and 0.3 µg/mL Thma α-L-fucosidase. With exception of Mfuc2, the double concentration of enzyme was used in assays with 1 mM substrate. All reactions were done at 30 °C.
