# Supplementary material for: Novel α-L-Fucosidases from a Soil Metagenome for Production of Fucosylated Human Milk Oligosaccharides
Source: PLoS One. 2016 Jan 22;11(1):e0147438. doi: 10.1371/journal.pone.0147438 (PMC4723247; doi:10.1371/journal.pone.0147438)
Supplement: S2 Fig — (PDF) [file pone.0147438.s002.pdf]

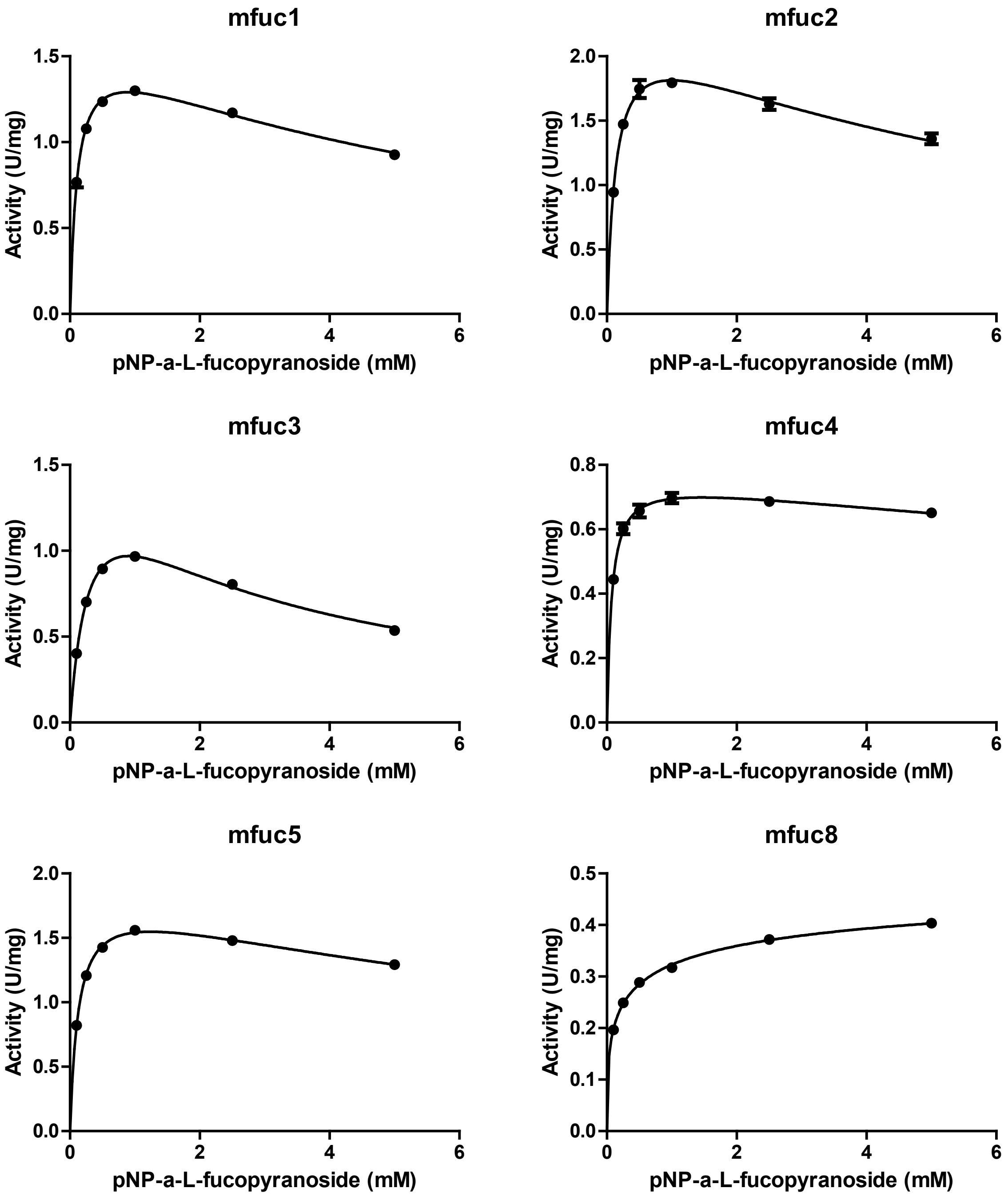
mfuc1
mfuc2
mfuc3
mfuc4
mfuc5
mfuc8
Activity (U/mg)
pNP-a-L-fucopyranoside (mM)
0.0
0.5
1.0
1.5
2.0
0.1
0.2
0.3
0.4
0.6
0.8
0
2
4
6

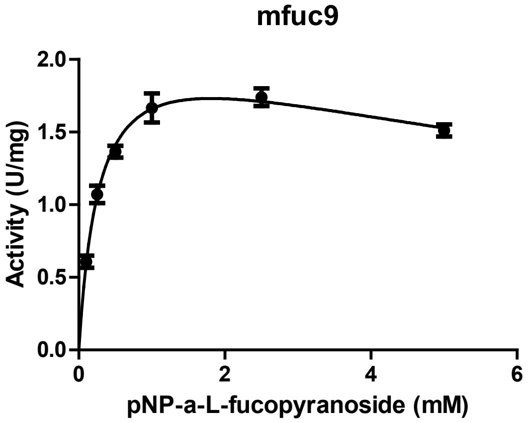

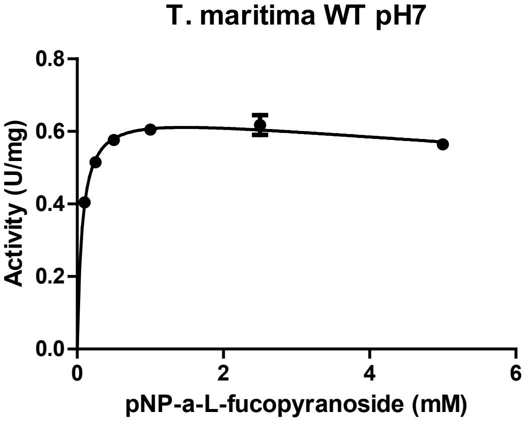

S2 Fig. Substrate concentration vs. hydrolase activity plots using pNP-Fuc as substrate. Substrate saturation curves for metagenomic α-L-fucosidases along with non-linear regression fits with sigmoidal allosteric (Mfuc8; $Y=V_{max}*X^h/(K_{prime} + X^h)$) and substrate inhibiton (all except Mfuc6, $Y=V_{max}*X/(K_m + X*(1+X/K_i))$) model. The various enzymes were assayed at 30 °C at their identified optimum pH. In case of of Thma WT, the optimum pH was 7 at 0.1 mM pNP-Fuc while two optima (pH 4 and 7) were observed at 1 mM pNP-Fuc, consequently substrate saturation was tested at both pH. Enzyme concentrations used were as follows: 0.2 µg/mL Mfuc1, 0.03 µg/mL Mfuc2, 0.6 µg/mL Mfuc3, 0.3 µg/mL Mfuc4, 0.2 µg/mL Mfuc5, 1.0 µg/mL Mfuc6, 0.2 µg/mL Mfuc7, 0.3 µg/mL Thma α-L-fucosidase. The concentrations pf pNP-Fuc tested were 0.1, 0.25, 0.5, 1.0, 2.5, and 5.0 mM
