# Supplementary material for: Novel α-L-Fucosidases from a Soil Metagenome for Production of Fucosylated Human Milk Oligosaccharides
Source: PLoS One. 2016 Jan 22;11(1):e0147438. doi: 10.1371/journal.pone.0147438 (PMC4723247; doi:10.1371/journal.pone.0147438)
Supplement: S3 Fig — (PDF) [file pone.0147438.s003.pdf]

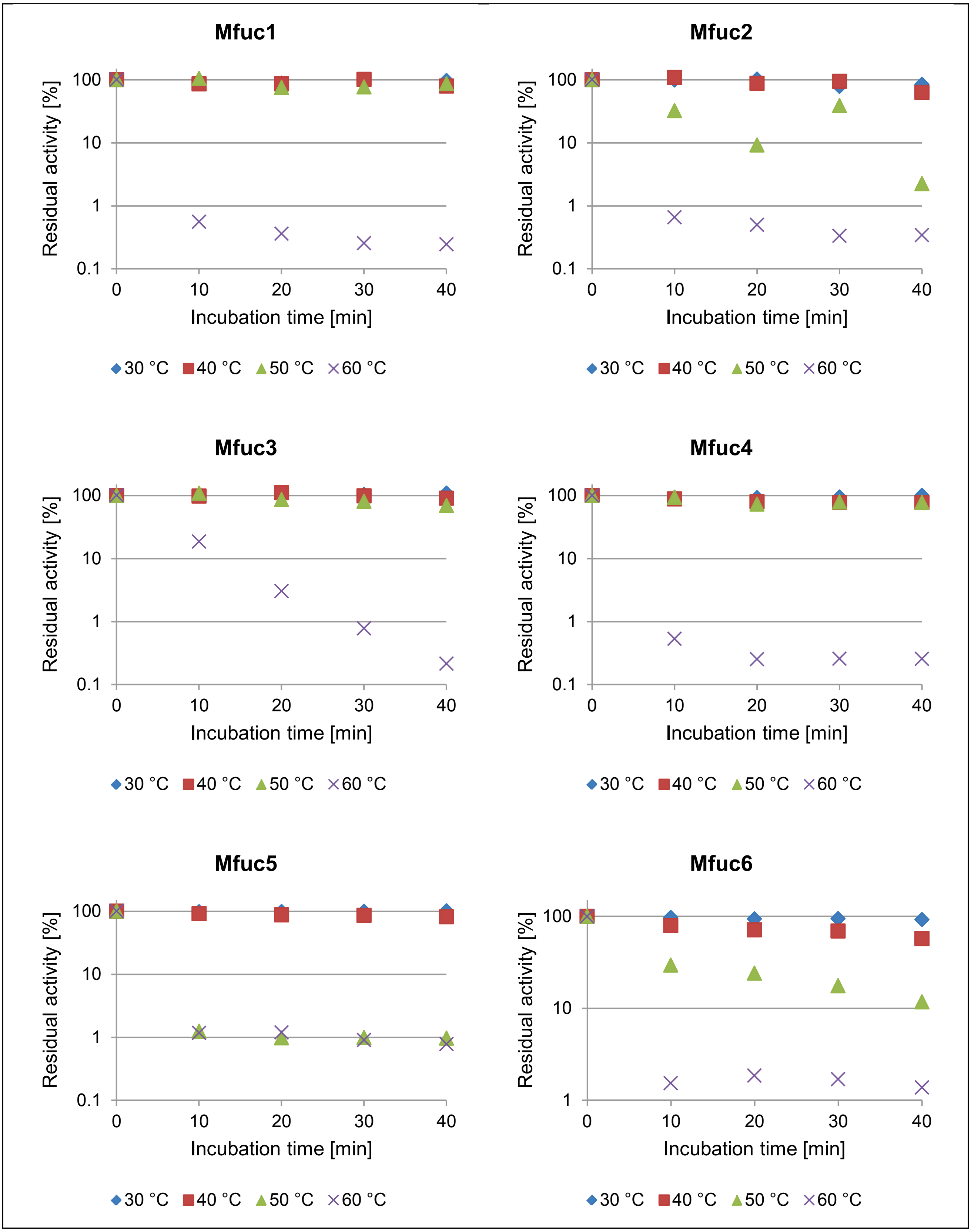
Mfuc1
Mfuc2
Mfuc3
Mfuc4
Mfuc5
Mfuc6
Residual activity [%]
100
10
1
0.1
0
10
20
30
40
Incubation time [min]
30 °C
40 °C
50 °C
60 °C

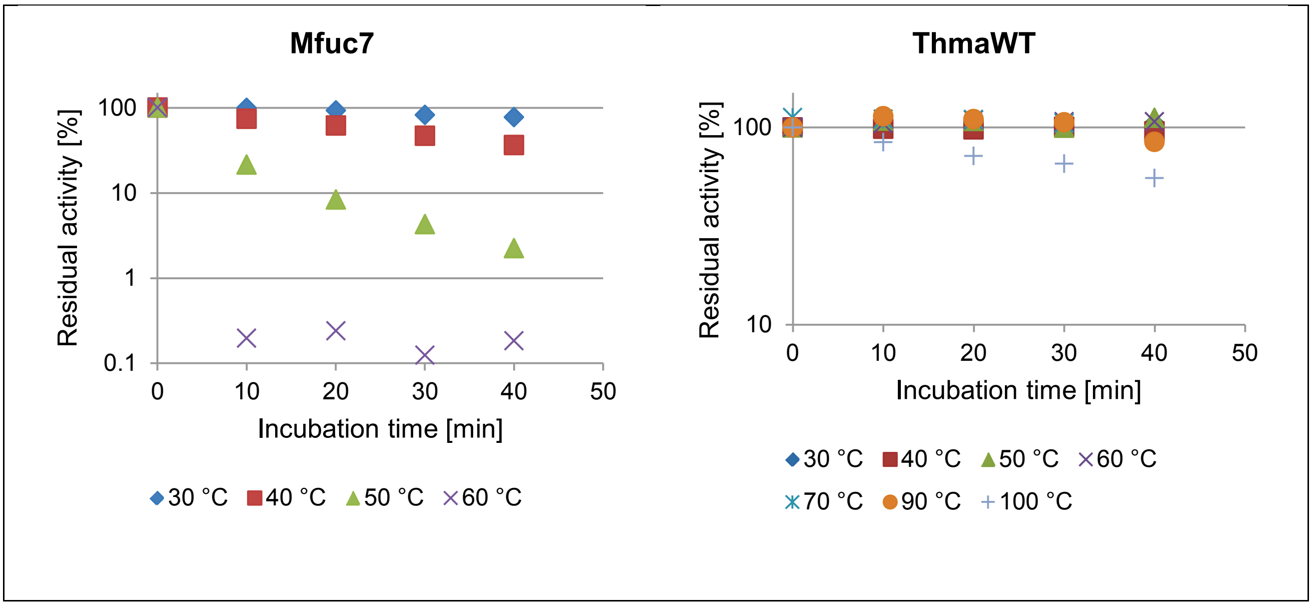

S3 Fig. Heat inactivation of α-L-fucosidases. The enzymes were diluted in a 5 mM pH buffer reflecting pH optimum and incubated at indicated temperatures and periods of time after which residual activity was measured in a continuous assay at pH 7 at 30 °C. Enzyme concentrations used in assays with 0.1 mM substrate were as follows: 0.8 µg/mL Mfuc1, 0.4 µg/mL Mfuc2, 1.2 µg/mL Mfuc3, 0.9 µg/mL Mfuc4, 0.7 µg/mL Mfuc5, 3.9 µg/mL Mfuc6, 0.5 µg/mL Mfuc7, 0.9 µg/mL Thma α-L-fucosidase. Activities were normalized on basis of enzyme activity with no heat treatment.
