# Supplementary material for: Novel α-L-Fucosidases from a Soil Metagenome for Production of Fucosylated Human Milk Oligosaccharides
Source: PLoS One. 2016 Jan 22;11(1):e0147438. doi: 10.1371/journal.pone.0147438 (PMC4723247; doi:10.1371/journal.pone.0147438)
Supplement: S4 Fig — (PDF) [file pone.0147438.s004.pdf]

```
                         10        20        30        40        50        60
                          |         |         |         |         |         |
Mfuc1            ------------MLKPKPTTGDTSWFIQDRFG--------------MFIHWGLYALP---
Mfuc4            ------------MYTPKPTQGDTTWFVHDRFG--------------MFIHWGLYSLP---
Mfuc7            ----------MTSYVTRKTPGDTAWFTHDRFG--------------MFIHWGLYAMP---
Mfuc2            -----------MQHQAAASTSDPAWFIHDRFG--------------LFIHWGIYSAA---
Mfuc5            -----------------MNNDRMQWFVDARFG--------------MFIHWGIYAIP---
Mfuc6            ------------------MKKTNTWFDSARFG--------------MFVHWGHCSQHGC-
Thma_opt         --MISMKPRYKPDWESLREHTVPKWFDKAKFG--------------IFIHWGIYSVPGWA
Mfuc3            METLPFRQVHLDFHTSPHIPAIGADFDPAQFAQSLQEARVNSISVFAKCHHGYSYYPTRV
                                          *   :*.                 * *

                         70        80        90       100       110       120
                          |         |         |         |         |         |
Mfuc1            ---------------------------SRHEWVQSQEKIAPEVYYEKYFKHFNP------
Mfuc4            ---------------------------SRHEWVQSQEQIPPET-YRQYFERFNP------
Mfuc7            ---------------------------GRHEWVKSRERIPEER-YDRYFRHFDP------
Mfuc2            ---------------------------ARHEWVKNLEKISDAD-YQPYFDHFDP------
Mfuc5            ---------------------------ARGEWVKQQESIVESA-YQPFFETFNP------
Mfuc6            ---------------------ELSWPLVGGVAALPEAKRLSVAEYHSTAATFNP------
Thma_opt         -----TPTGELG----KVPMDAWFFQNPYAEWYENSLRIKESPTWEYHVKTYGENFEYEK
Mfuc3            GTPHPHLQRDLLGEMVEACHSAGIRVQAYTTVVWDELAWAEHPEWRQIYPDGRLGRSGGP
                                                :

                        130       140       150       160       170       180
                          |         |         |         |         |         |
Mfuc1            -----------TKYDPRLWARAAREAGMK----YVVLTTKHHEGFCLWDSQFTD-YKVT-
Mfuc4            -----------TLYDPRKWARAAREAGMK----YVVLTTKHHEGFCLWDSAYTD-YKAS-
Mfuc7            -----------DLYDAGEWARQARDAGMK----YVVMTTKHHEGFCMFDSRYTD-YKCT-
Mfuc2            -----------DLYDPAAWARDARNAGMK----YVVVTTKHHDGFCLWDSALTD-YKAP-
Mfuc5            -----------YNFDPAEWAKAARKAGMK----YAVMTSKHHDGFCLFDSQYTD-YKAT-
Mfuc6            -----------QGYDPKEWARLAKRVGMQ----YAIFTSKHHDGYAMYHTAEND-FSIG-
Thma_opt         FADLF----TAEKWDPQEWADLFKKAGAK----YVIPTTKHHDGFCLWGTKYTD-FN---
Mfuc3            LKPDWKNLCPNTSYSDYVIAQIEEVLDLYPVDGIFIDIVRYIGGPCICTTCLRQMQAEGV
                              :.    *   .  .        :   ::  *  :  :   :

                        190       200       210       220       230       240
                          |         |         |         |         |         |
Mfuc1            -NTPYG----------KDLLTPFVEAFRAEGIRVGFYYS---IIDWHHPEFPVD--VFHP
Mfuc4            -NTPYG----------KDLLKPFVEAFRAEGLHVGFYYS---IIDWHHPDFLID--VFHP
Mfuc7            -NTPAG----------RDLVREYADAFRAEGLRVGFYYS---LIDWHHPDFPID--MLHP
Mfuc2            -NTPAG----------RDLLRPLVEAFRAEGLKIGFYHS---LLDWHHPDFPVD--GLHP
Mfuc5            -NTPAG----------RDLVREYVDAFRAEGLKVGFYYS---LLDWHHPDYPIAGDSIHP
Mfuc6            -HSPYG----------KDIVRSFLDAMRAEGLRVGIYFS---LIDWHHPDYPAFTDADRP
Thma_opt         -SVKRG--P------KRDLVGDLAKAVREAGLRFGVYYSG--GLDWRFTTEPIR------
Mfuc3            DPQDKAQLYRFSLAAERRFMRRTTEAIRAKREGVAIFYNSRLRVEWDEKMGNRPELDDFT
                      .          : ::    .*.*     ...:..    ::*

                        250       260       270       280       290       300
                          |         |         |         |         |         |
Mfuc1            LRDHPDVAEINQGR-DVRRYAEYLRNQVEELLTRY-NPEILWCDFS------------YP
Mfuc4            LRNHPEVARLNEGR-DMRRYAAYMRNQVTELLTNYGDIDIIWFDFS------------YP
Mfuc7            RREDTDAREMSHGR-DIRKYACYMRDQVRELLTNYGKIDILWFDFS------------YG
Mfuc2            MRDDLAFREAAQGR-DMARYREYLFGQTRELLTQFGKIDIMWFDFS------------YS
Mfuc5            MRNHPDFQNHTG---NLPRYADYVRDQVRELLTNYGMIDIIWFDFS------------YG
Mfuc6            YRWGQWRRSSPE---AWVRYQQFMFGQIRELLTEYGKIDIIWFDG---------------
Thma_opt         --YPEDLSYIRP---NTYEYADYAYKQVMELVDLY-LPDVLWNDMG------------WP
Mfuc3            HLEIESLPGGFWGYSHFPLNVRYFQTYDRPLIGMTGRFHTTWGDFGGLRNRAALEFECFQ
                                      :       *:         * *
```

```
                    310       320       330       340       350       360
                      |         |         |         |         |         |
Mfuc1        HATYKG----LPGKGRNDWQSESLVAMIRAISPKI------ILNNRLDLP--------IA
Mfuc4        GRDYNG----LPGKGRNEWESEKLYALVRRLRPHI------IIDNRLDLPA-------HL
Mfuc7        SWTGEGDKAWMKGKGKDDWESEELIRIARELQPGI------IIDNRTEIE---------Q
Mfuc2        ELNWGWS----KGRGKADWHSEELMAMVRELQPGI------LLNDRLEVG----------
Mfuc5        EMRGEA------------WKATELVKMVRELQPGI------IIDNRLGGDIKAAEPEIYA
Mfuc6        GWERTP----------DEWKATELEAMIRSLQPEI------LINDRLPGA----------
Thma_opt     EKGKED--------------LKYLFAYYYNKHPEG------SVNDRWG------------
Mfuc3        ALAQGAGCSIGDQLHPRGQLDPAVYQRIGEVYAEVERREAWVQNTRPLPEVGVLTVNGAP
                                    :        .          : *

                    370       380       390       400       410       420
                      |         |         |         |         |         |
Mfuc1        AD-----IYTPEQVQPTEWVQVD---GEPVVWEACQTFSGSWGYHRDE--ETWKSPEQLI
Mfuc4        AD-----VHTPEQWQPTEWVKVD---GEPVVWEACQTFSGSWGYHRDE--TTWKDAGQLI
Mfuc7        D------LWTPEQYQPTEWVRHPQ-TGELVVWEACQTLSGSWGYHRDE--QTWKSPEMLL
Mfuc2        GD-----IVTPEQYQPHGWMKVN---GQRVTWEACQTFNGSWGYHRDN--LDWKPAGMLV
Mfuc5        GD-----FASPEQIIPPEGVVNEL--GAPVPWEACITLNDHWGYAAWD--KNWKSPKTVV
Mfuc6        GD-----YDTPEQFVPPQLPARA----W----EACMTINESWGYNPDD--KQFKSGRQLV
Thma_opt     --------VPHWDFKTAEYHVNYPGDLPGYKWEFTRGIGLSFGYNRNEGPEHMLSVEQLV
Mfuc3        GVSGDMLHSAGDSLPLSDLGALFALEQQKVQFQFLDASNDLSPYQVVILPDQVAVDDRLA
                         .                   :     .    *              :
                    430       440       450       460       470       480
                      |         |         |         |         |         |
Mfuc1        MMLVNTVALGGNLLMN--------------------------------VGPTSLGTLDGR
Mfuc4        RMLVNTVARGGNLLMN--------------------------------VGPTSRGTFDQR
Mfuc7        RMLVNTVSLGGNLLMN--------------------------------VGPTSRGYLDAR
Mfuc2        QMLIDSVSKGGNFLLN--------------------------------VGPNGRGEFEPR
Mfuc5        RALVECVSKGGNLLLN--------------------------------VGPTAKGEIPAE
Mfuc6        HTLCEIAARGGNLLLN--------------------------------VGPMADGKLPSE
Thma_opt     YTLVDVVSKGGNLLLN--------------------------------VGPKGDGTIPDL
Mfuc3        ERLRAYLRAGGKLLVSDRSGLQDGDFTLAEEMGVHYAGPAPYAPDYLLLEPEISAGIELM
               :      .*::*:.                                : *   . :

                    490       500       510       520       530       540
                      |         |         |         |         |         |
Mfuc1        ALKALGVYRDWMALHSESIYGCTQSE-----FPTPQDCRLTQ------------------
Mfuc4        ALDALAVYRDWMAVHSESIYGCTQSA-----YQEPLDCRLTQ------------------
Mfuc7        AETALKAYAGWMRYNSRSIYGCTMAEPEFL-SAAPPDCRLTQSM----------------
Mfuc2        AVERLRQIGAWMHLHRRAITGCTASQ-----FTPPPDGRYTQ------------------
Mfuc5        CLTILDQVGGWMHANGTSVYACGSTD-----LPKPEWGRFTR------------------
Mfuc6        LLERLAVIEGWMARNAESIIGTAPGL-----EPWQFYGPSTR------------------
Thma_opt     QKERLLGLGEWLRKYGDAIYGTSVWERCCAKTEDGTEIRFTR------------------
Mfuc3        AHSCEQQGSRVILASGARVLARSGAPYFNRTWDHFCSHQYTPMAEDSGDPVAVEHGNVIY
                        :      :                     *

                    550       560       570       580       590       600
                      |         |         |         |         |         |
Mfuc1        -----------NGKKLYVHVYAWPFK-FLQLQGLGG-----------KAEFARFLHDGSE
Mfuc4        -----------NGNKLYVHIFAWPFR-HLYLEGLAD-----------KVAYARFLHDGSE
Mfuc7        -----------DGKRLYVHLYAYPFA-HLQLKGLAG-----------RVEYAQFLHDGSE
Mfuc2        -----------KGKRLYLHLFAWPFR-HVHLEGLAG-----------RVEYAQLLSDGSE
Mfuc5        -----------RGDRLYAHILDRGIG-PVNLRRLQD-----------RLVYARLLADDSE
Mfuc6        -----------RDDRIYLHLLMRPYD-TVSVRGLPIR----------RVRSVRALGADRE
Thma_opt     -----------KCNRIFVIFLGIPTGEKIVIEDLNLS----------AGTVRHFLTGER-
Mfuc3        LARPLFREYAISAKRVHKQVIGNCLARLLPAPRVGANNLPSTAIVTVRQQGADLLVHLLH
                            :.  .        :    :                    *
```

```
                     610       620       630       640       650       660
                       |         |         |         |         |         |
Mfuc1         VQMDL----PEWEAGQLSVADDTLLLHLPIK-KPDAVVPVIELTLK--------------
Mfuc4         IVLKP----TAWYSDHLTRGENTLVLELPVK-QPNTIVPVIELELKE-------------
Mfuc7         VKLIEGA--VKHYGEGVPTSEDMLELEIPPV-KPASLLPVIELFLK--------------
Mfuc2         IKRIELDPAVKASTIHMTGKPGTLTLELPVQ-RPDVLVPVIELFLK--------------
Mfuc5         IKLAR-----PWNSAEYP--DDAFITFPTAA-LPDDWDTVVELVLK--------------
Mfuc6         LAFTTRAPIIESLFGADPVGEITITVPEDVV-DPYATVLAIDVA----------------
Thma_opt      LSFKNVGKNLEITVP------KKLLETDSIT-LVLEAVEE--------------------
Mfuc3         YVHQRRGQGLDVIEDALPLVDIELSVRAECRPQTVQLVPELQPVEWSWQEGYVHLRIPRI
                                     :

                     670
                       |
Mfuc1         --------------
Mfuc4         --------------
Mfuc7         --------------
Mfuc2         --------------
Mfuc5         --------------
Mfuc6         --------------
Thma_opt      --------------
Mfuc3         NGYQIVQLQGAARE
```

S4 Fig. Multiple sequence alignment of α-L-fucosidases reported in this study. The alignment was done using ClustalW. The catalytic nucleophile aspartate that is conserved in GH-29 family is shown with yellow background.
