# Supplementary material for: Novel α-L-Fucosidases from a Soil Metagenome for Production of Fucosylated Human Milk Oligosaccharides
Source: PLoS One. 2016 Jan 22;11(1):e0147438. doi: 10.1371/journal.pone.0147438 (PMC4723247; doi:10.1371/journal.pone.0147438)
Supplement: S5 Fig — (PDF) [file pone.0147438.s005.pdf]

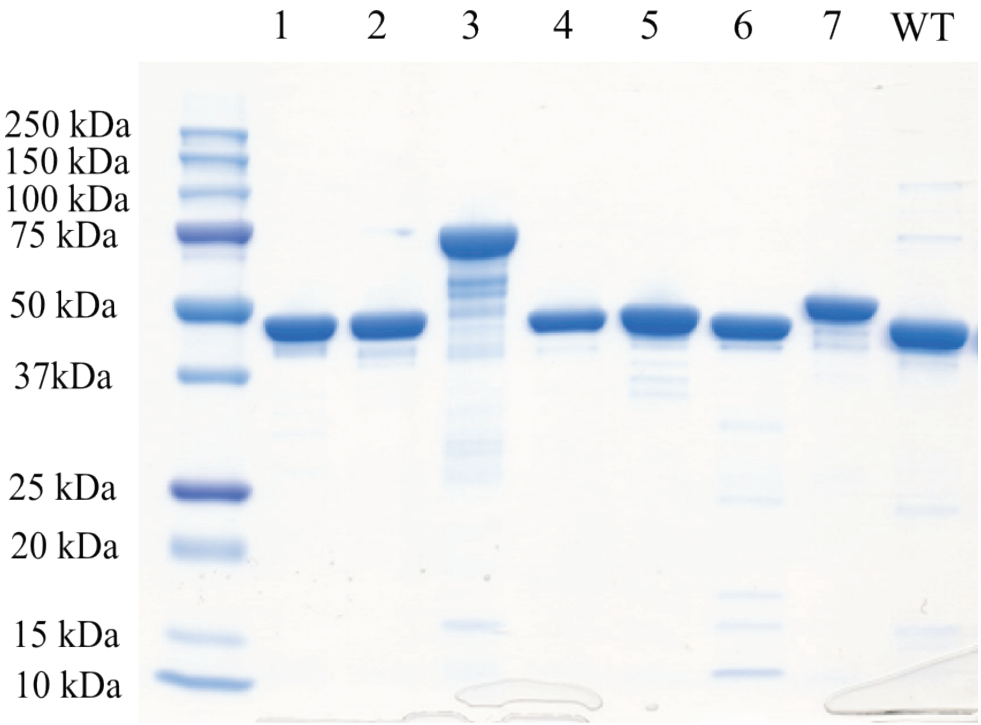

S5 Fig. SDS-PAGE of recombinant Mfuc1-7 and Thma. The first lane contains a molecular size marker, Mfuc1-7 is in the lanes denoted 1-7 respectively, while Thma is denoted WT. In all of the preparations the prominent band corresponds to the theoretical size of the respective enzymes. As is commonly observed in IMAC purification of His-tagged proteins from *E. coli* lysates, small amounts of contaminating *E. coli* proteins are observed.
