# Supplementary material for: Novel α-L-Fucosidases from a Soil Metagenome for Production of Fucosylated Human Milk Oligosaccharides
Source: PLoS One. 2016 Jan 22;11(1):e0147438. doi: 10.1371/journal.pone.0147438 (PMC4723247; doi:10.1371/journal.pone.0147438)
Supplement: S6 Fig — (PDF) [file pone.0147438.s006.pdf]

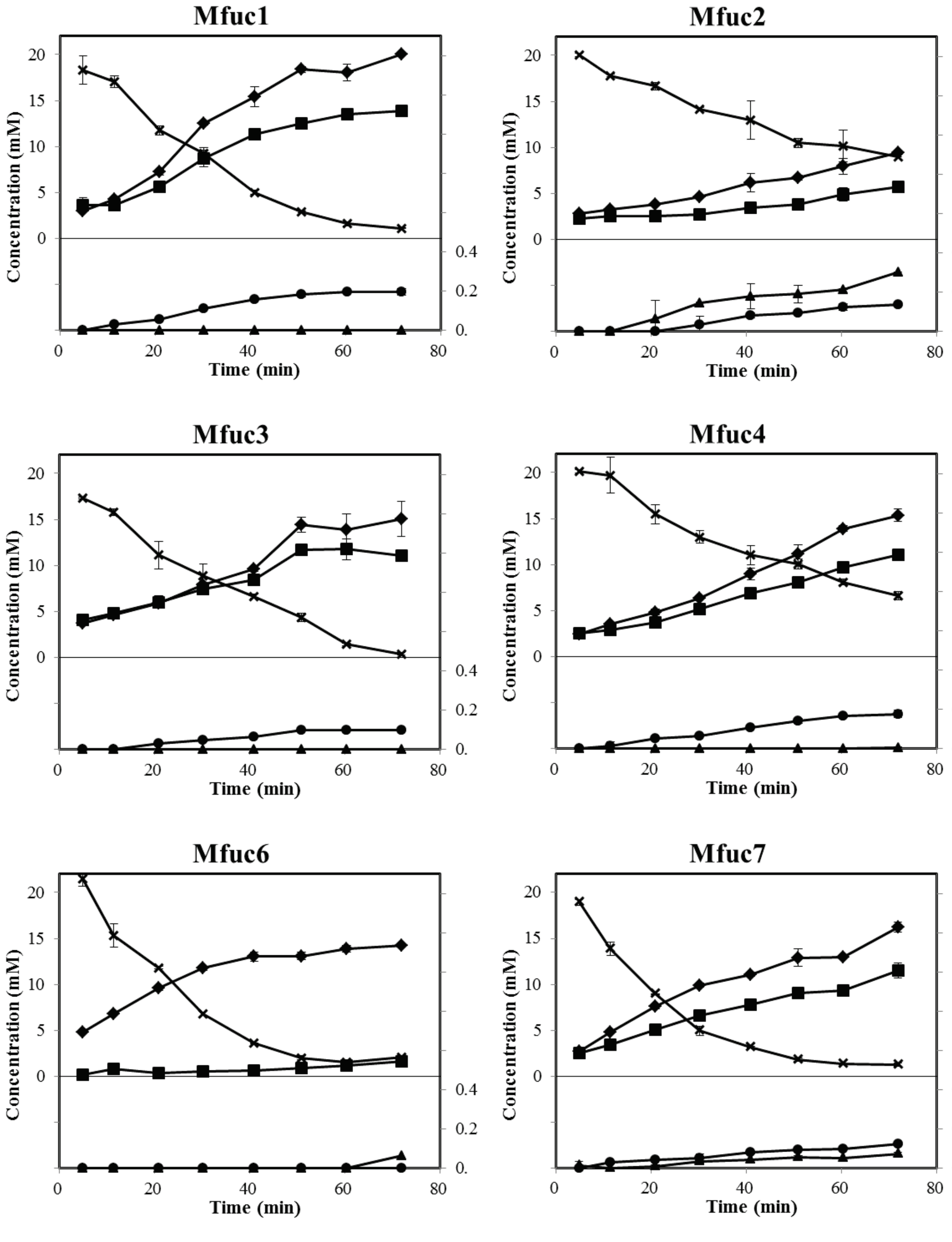

S6 Fig. Time study of trans-fucosylation catalysed by metagenome-derived α-L-fucosidases. Reactions were done using 25 mM pNP-Fuc as donor and 100 mM lactose as acceptor at 30 °C and optimal pH. The concentrations were determined by HPAEC-PAD and for pNP by spectrophotometry. L-fucose (■), pNP (♦), pNP-Fuc (X), 2'-FL (▲), and FL (●). Note that the concentrations on the secondary axis are lower than for Thma and Mfuc5 in the main text.
