# Supplementary material for: Novel α-L-Fucosidases from a Soil Metagenome for Production of Fucosylated Human Milk Oligosaccharides
Source: PLoS One. 2016 Jan 22;11(1):e0147438. doi: 10.1371/journal.pone.0147438 (PMC4723247; doi:10.1371/journal.pone.0147438)
Supplement: S1 File — (PDF) [file pone.0147438.s007.pdf]

S1 File. Sequence of codon-optimised Thma-encoding gene. Gene sequence in FASTA format

```
>Thma

ATGATTTCAATGAAACCGCGTTATAAACCGGATTGGGAATCACTGAGAGAACATACAGTTCCGAAATGGTTTG
ATAAAGCGAAATTTGGCATTTTTATCCATTGGGGCATCTATTCAGTTCCGGGATGGGCAACACCGACAGGCGA
ACTGGGCAAAGTTCCGATGGATGCATGGTTTTTTCAAAATCCGTATGCGGAATGGTATGAAAATAGCCTGCGC
ATTAAAGAATCACCGACATGGGAATATCATGTCAAAACATATGGCGAAAACTTTGAATATGAAAAATTTGCGG
ATCTTTTTACAGCGGAAAAATGGGATCCGCAAGAATGGGCAGATTTGTTCAAAAAAGCAGGCGCAAAATATGT
CATCCCGACAACAAAACATCATGATGGCTTTTGCCTGTGGGGCACGAAGTATACAGATTTTAACAGCGTCAAA
CGCGGACCGAAAAGAGATCTGGTTGGCGATCTGGCAAAAGCAGTTAGAGAAGCAGGCCTGAGATTTGGCGTCT
ATTATTCAGGCGGACTGGATTGGAGATTTACAACAGAACCGATTCGCTATCCGGAAGATCTGTCATATATTAG
ACCGAACACATATGAATATGCGGATTATGCGTATAAACAGGTTATGGAACTGGTCGATCTGTATCTGCCGGAT
GTTCTGTGGAATGATATGGGCTGGCCTGAAAAAGGCAAAGAAGATCTGAAATATCTTTTTGCGTATTACTATA
ACAAACATCCGGAAGGCAGCGTCAATGATAGATGGGGAGTTCCGCATTGGGATTTCAAAACAGCAGAATATCA
TGTGAACTATCCTGGCGATCTTCCGGGATATAAATGGGAATTTACAAGAGGCATTGGCCTTAGCTTTGGCTAT
AATAGAAATGAAGGACCGGAACATATGCTGTCAGTTGAACAACTGGTTTATACACTGGTTGATGTTGTTAGCA
AAGGCGGAAATCTGCTGCTGAATGTTGGACCGAAAGGCGATGGCACAATTCCGGATCTGCAAAAAGAAAGACT
GCTGGGCCTGGGCGAATGGCTGAGAAAATATGGAGATGCAATTTATGGCACAAGCGTTTGGGAAAGATGCTGC
GCAAAAACAGAAGATGGCACAGAAATTCGCTTTACAAGAAAATGCAACCGCATTTTTGTCATCTTTCTGGGCA
TTCCTACAGGCGAAAAAATTGTTATTGAAGATCTTAATCTGAGCGCAGGCACAGTTCGCCATTTTCTGACAGG
CGAAAGACTGAGCTTTAAAAACGTTGGCAAAAACCTGGAAATCACGGTTCCGAAAAAACTGCTGGAAACGGAT
TCAATTACACTGGTTCTGGAAGCAGTCGAGGAA
```
