# Supplementary material for: Novel α-L-Fucosidases from a Soil Metagenome for Production of Fucosylated Human Milk Oligosaccharides
Source: PLoS One. 2016 Jan 22;11(1):e0147438. doi: 10.1371/journal.pone.0147438 (PMC4723247; doi:10.1371/journal.pone.0147438)
Supplement: S2 File — (PDF) [file pone.0147438.s008.pdf]

S2 File. Immuno-glycan microarray analysis of fucosylated xyloglucan cleavage

Method

The extraction of cell wall components and subsequent analysis was performed essentially as previously reported [1]. 4 M NaOH (containing 0.1 % $NaBH_4$) extracts from the different plant species were spotted using a microarray robot (Sprint, Arrayjet, Roslin, UK). Each extracted sample was printed as a two-fold dilution followed by 3 five-fold dilutions. All sample dilutions were performed in printing buffer (55.2 % glycerol, 44 % water, 0.8 % Triton X-100). The samples were printed onto nitrocellulose membranes with a pore size of 0.45 µm (Whatman, Maidstone, UK). Once printed, arrays were blocked with PBS (140 mM NaCl, 2.7 mM KCl, 10 mM Na_2HPO_4, 1.7 mM KH_2PO_4, pH 7.5) containing 5 % w/v low fat milk powder (M-PBS) for 1 h. Hereafter, they were washed in PBS and incubated with or without 10 µg α-L-fucosidases at 30 °C at optimal pH. After incubation, arrays were washed thoroughly with PBS, followed by 2 h incubation with antibodies: 10-fold diluted LM15 (PlantProbes, Leeds University, UK) or 250-fold diluted CCRC-M1 (Complex Carbohydrate Research Center) in M-PBS. LM15 is monoclonal antibody specific for xyloglucan epitopes XXX(G) and XLX(G)/XXL(G) that are not attacked by α-L-fucosidases [2,3] and antibody CCRC-M1 is specific for fucosylated xyloglucan [4]. Subsequently, arrays were washed in PBS and incubated with anti-rat or anti-mouse secondary antibody respectively conjugated to alkaline phosphatase (Sigma, Poole, UK) diluted 1/5000 in M-PBS. After washing with PBS and distilled H_2O, arrays were developed in a solution containing 5-bromo-4-chloro-3-indolylphosphate and nitro blue tetrazolium in alkaline phosphatase buffer (100 mM NaCl, 5 mM $MgCl_2$, 100 mM diethanolamine, pH 9.5). Developed microarrays were scanned at 2400 dpi (CanoScan 8800F, Canon, Søborg, Denmark) and converted to TIFFs. Antibody signals were measured using appropriate software (Array-Pro Analyzer 6.3, Media Cybernetics, Rockville, USA). Data were presented as two datasets where maximal spot signal was set to 100 and all other values normalized accordingly. Color intensity is correlated to mean spot signal value and a cut-off of 5 was applied.

Results

		pH 7						pH 9		pH 6		
		Control	Thma	Mfuc1	Mfuc2	Mfuc4	Mfuc5	Control	Mfuc6	Control	Mfuc3	Mfuc7
Arabidopsis thaliana	**Xyloglucan control (LM15)**	23	21	23	22	20	21	21	22	23	23	24
	**Fucosylated xyloglucan (CCRC-M1)**	32	28	0	5	7	0	29	27	28	26	16
Sambucus nigra	**Xyloglucan control (LM15)**	89	92	100	95	90	88	88	98	89	91	99
	**Fucosylated xyloglucan (CCRC-M1)**	99	90	0	15	9	0	91	86	100	93	56

Fig. Immuno-glycan micro-array of cell walls from selected plant species (Arabidopsis thaliana and Sambucus nigra). Release of fucose from xyloglucans was tested by seven new α-L-fucosidases (Fuc1-7), and Thma. Xyloglucan was measured with the antibody LM15, while fucosylated xyloglucan was measured using the antibody CCRC-M1. After incubation with the α-L-fucosidases differential decline of the fucosylated xyloglucan CCRC-M1 epitopes were observed with Mfuc1, 2, 4, 5 and 7.

References

1. Moller I, Sørensen I, Bernal AJ, Blaukopf C, Lee K, Øbro J, et al. High-throughput mapping of cell-wall polymers within and between plants using novel microarrays. The Plant Journal. 2007;50: 1118-1128.
2. Pedersen HL, Fangel JU, McCleary B, Ruzanski C, Rydahl MG, Ralet MC, et al. Versatile high resolution oligosaccharide microarrays for plant glycobiology and cell wall research. J. Biol. Chem. 2012;287: 39429-39438.
3. Marcus SE, Verhertbruggen Y, Herve C, Ordaz-Ortiz JJ, Farkas V, Pedersen HL, et al. Pectic homogalacturonan masks abundant sets of xyloglucan epitopes in plant cell walls. BMC Plant. Biol. 2008;8: 60.
4. Puhlmann J, Bucheli E, Swain MJ, Dunning N, Albersheim P, Darvill AG, Hahn MG. 1994. Generation of monoclonal antibodies against plant cell-wall polysaccharides. I. Characterization of a monoclonal antibody to a terminal alpha-(1-->2)-linked fucosyl-containing epitope. Plant Physiol. 1994;104: 699-710.
