# Supplementary material for: Novel α-L-Fucosidases from a Soil Metagenome for Production of Fucosylated Human Milk Oligosaccharides
Source: PLoS One. 2016 Jan 22;11(1):e0147438. doi: 10.1371/journal.pone.0147438 (PMC4723247; doi:10.1371/journal.pone.0147438)
Supplement: S3 File — (PDF) [file pone.0147438.s009.pdf]

S3 File. Selected NMR spectra, NMR chemical shifts and comparison of ^{1}H NMR integrals

This file contains:

Figure I. ^{1}H NMR spectrum (D_2O, 400 MHz) of *p*-nitrophenyl α-L-fucose.

Figure II. ^{1}H NMR spectrum (D_2O, 500 MHz) of post-enzymatic reaction mixture by Mfuc1 enzyme with expansion of the α-anomeric region.

Figure III. ^{1}H NMR spectrum (D_2O, 500 MHz) of post-enzymatic reaction mixture by Mfuc2 enzyme with expansion of the α-anomeric region.

Figure IV. ^{1}H NMR spectrum (D_2O, 500 MHz) of post-enzymatic reaction mixture by Mfuc3 enzyme with expansion of the α-anomeric region.

Figure V. ^{1}H NMR spectrum (D_2O, 500 MHz) of post-enzymatic reaction mixture by Mfuc4 enzyme with expansion of the α-anomeric region.

Figure VI. ^{1}H NMR spectrum (D_2O, 500 MHz) of post-enzymatic reaction mixture by Mfuc5 enzyme with expansion of the α-anomeric region.

Figure VII. ^{1}H NMR spectrum (D_2O, 500 MHz) of post-enzymatic reaction mixture by Mfuc6 enzyme with expansion of the α-anomeric region.

Figure VIII. ^{1}H NMR spectrum (D_2O, 500 MHz) of post-enzymatic reaction mixture by Mfuc7 enzyme with expansion of the α-anomeric region.

Figure IX. ^{1}H NMR spectrum (D_2O, 500 MHz) of post-enzymatic reaction mixture by Thma with expansion of the α-anomeric region.

Figure X. ^{1}H NMR spectra (D_2O, 400 MHz) of individual self-condensation products separated by HPLC and comparison with products of selected enzymatic reactions.

NMR chemical shifts of selected compounds characterized

Figure XI. Comparison of ^{1}H NMR integrals for reaction mixtures of the different enzymes.

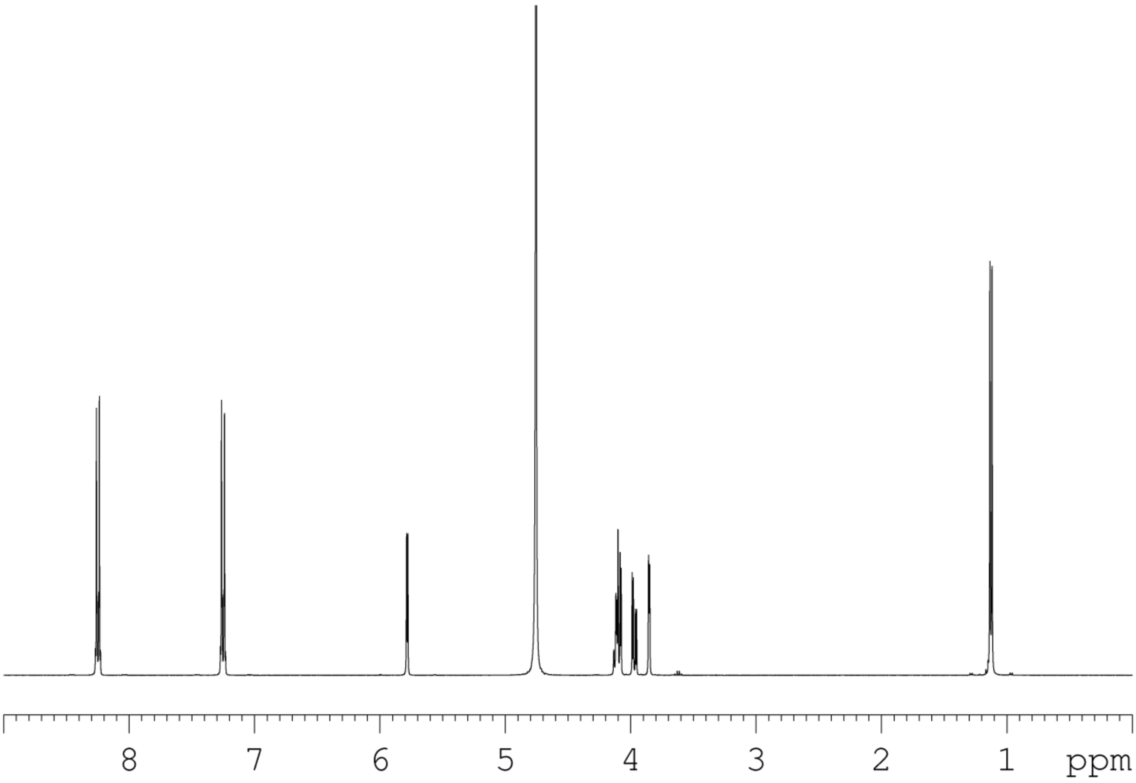

Figure I. ^{1}H NMR spectrum (D_2O, 400 MHz) of *p*-nitrophenyl α-L-fucose.

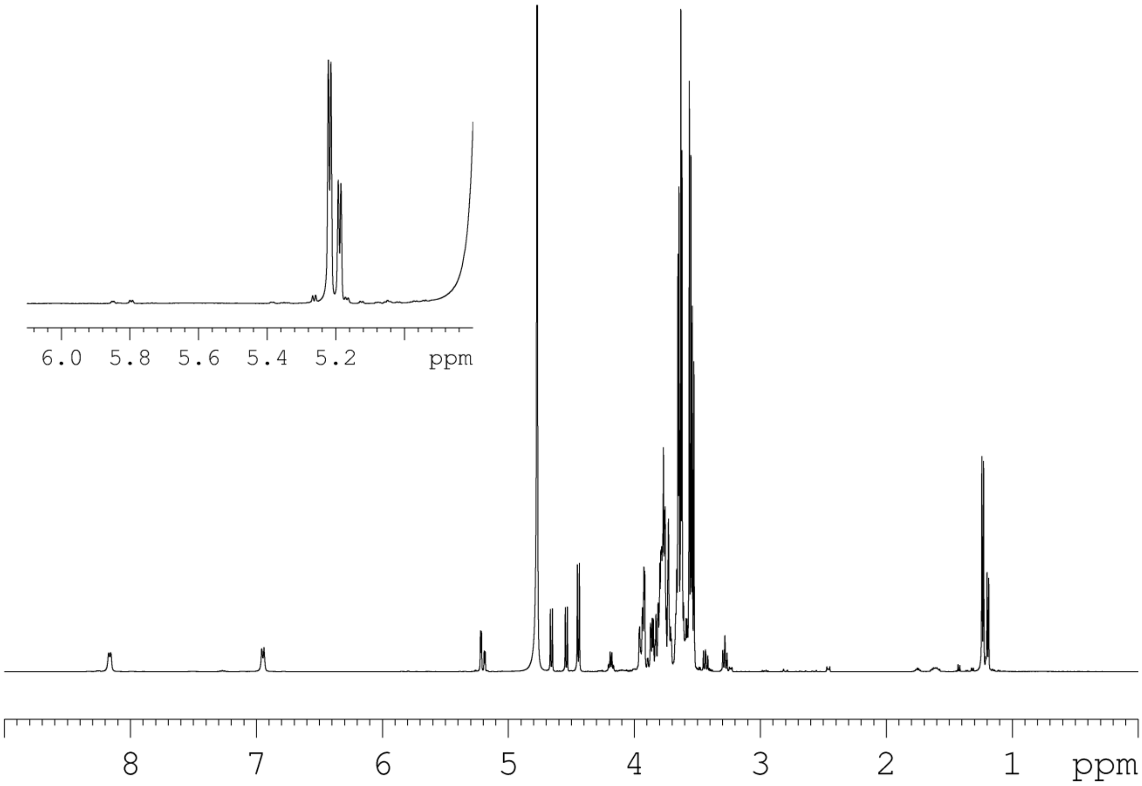

Figure II. ^{1}H NMR spectrum (D_2O, 500 MHz) of post-enzymatic reaction mixture by Mfuc1 enzyme with expansion of the α-anomeric region.

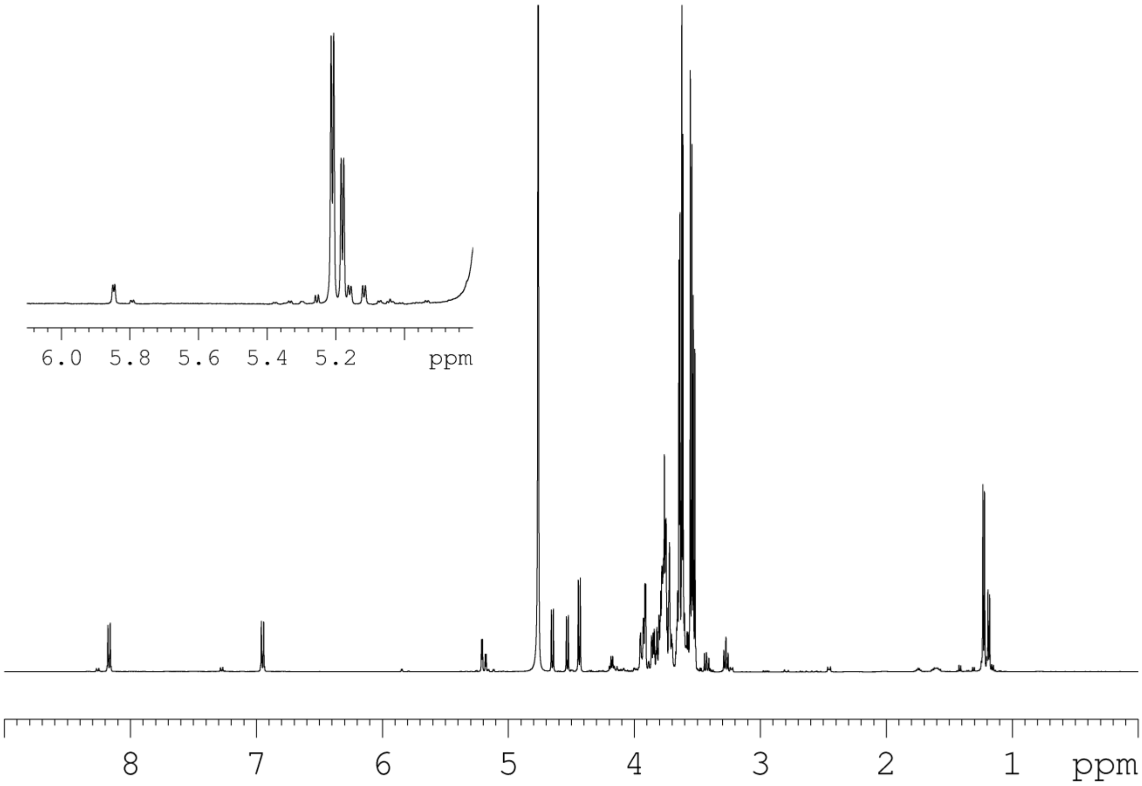

Figure III. ^{1}H NMR spectrum (D_2O, 500 MHz) of post-enzymatic reaction mixture by Mfuc2 enzyme with expansion of the α-anomeric region.

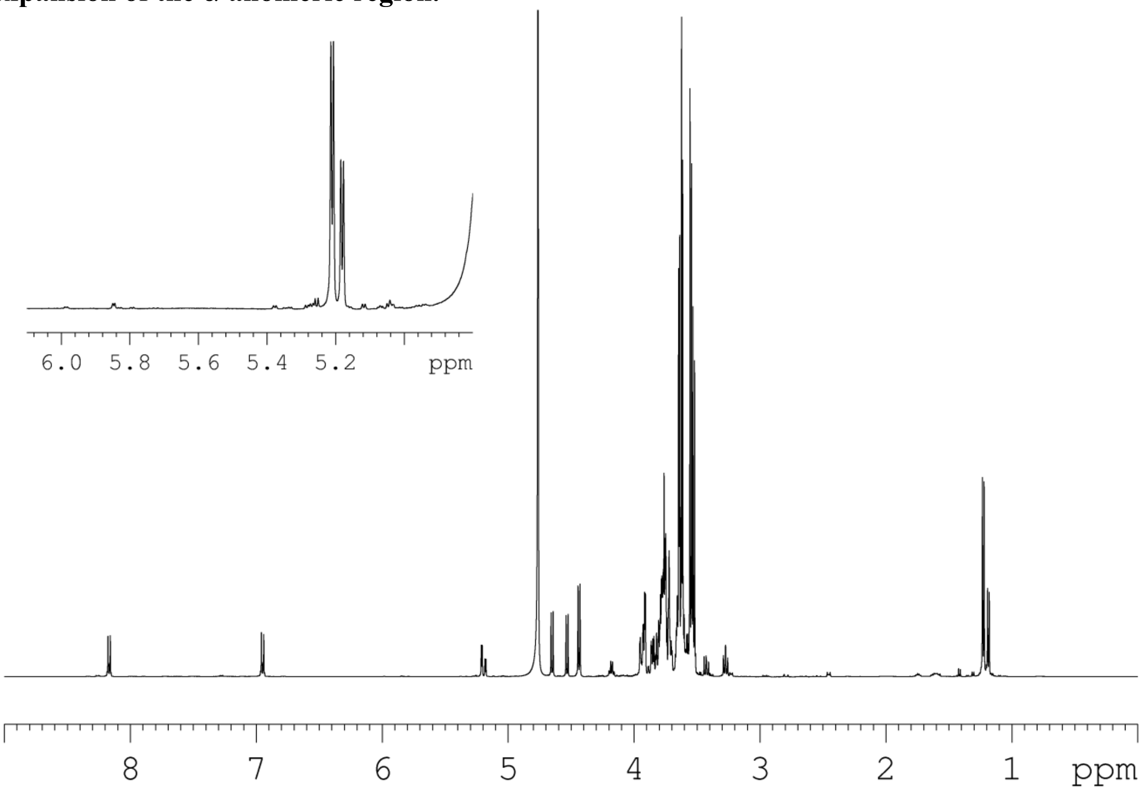

Figure IV. ^{1}H NMR spectrum (D_2O, 500 MHz) of post-enzymatic reaction mixture by Mfuc3 enzyme with expansion of the α-anomeric region.

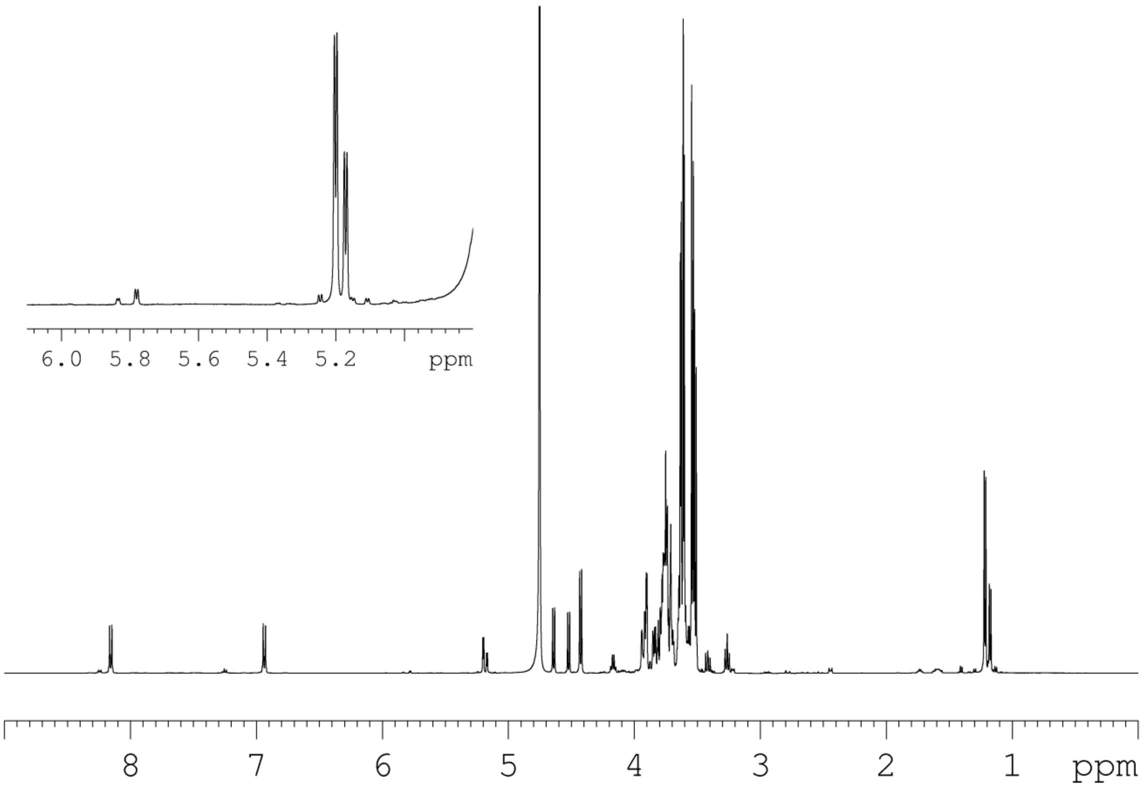

Figure V. ^{1}H NMR spectrum (D_2O, 500 MHz) of post-enzymatic reaction mixture by Mfuc4 enzyme with expansion of the α-anomeric region.

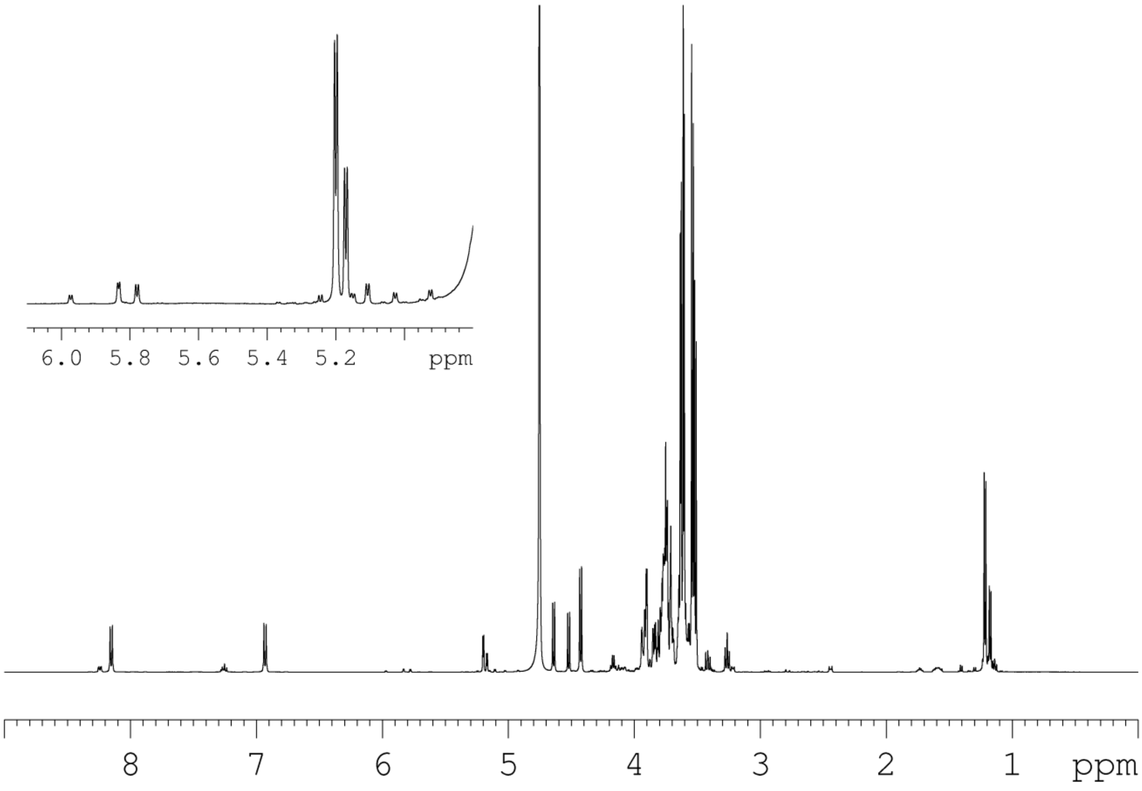

Figure VI. ^{1}H NMR spectrum (D_2O, 500 MHz) of post-enzymatic reaction mixture by Mfuc5 enzyme with expansion of the α-anomeric region.

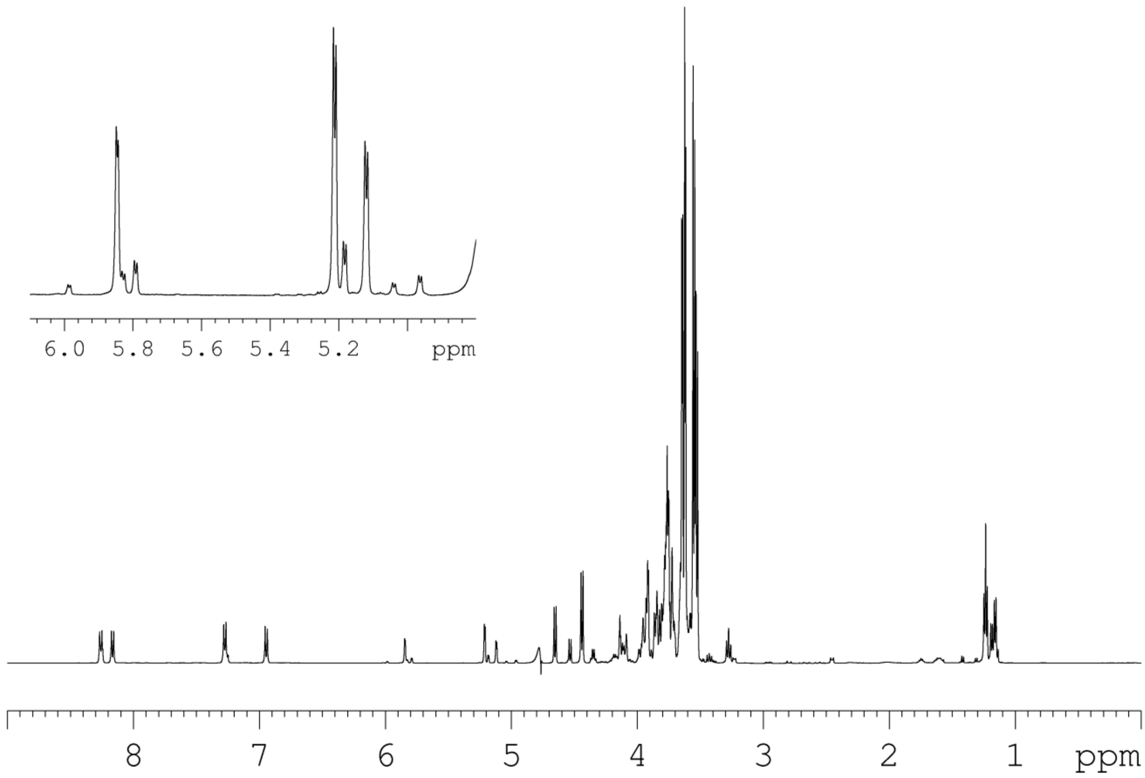

Figure VII. ^{1}H NMR spectrum (D_2O, 500 MHz) of post-enzymatic reaction mixture by Mfuc6 enzyme with expansion of the α-anomeric region. The residual solvent signal was removed by presaturation.

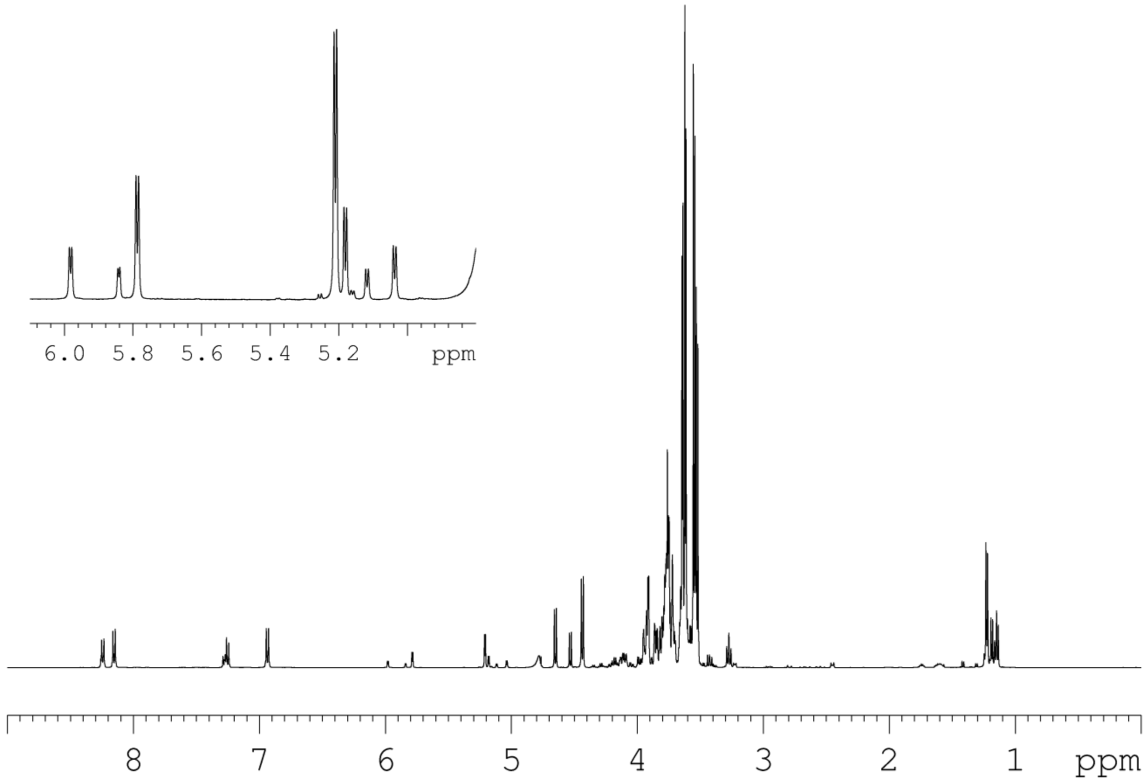

Figure VIII. ^{1}H NMR spectrum (D_2O, 500 MHz) of post-enzymatic reaction mixture by Mfuc7 enzyme with expansion of the α-anomeric region. The residual solvent signal was removed by presaturation.

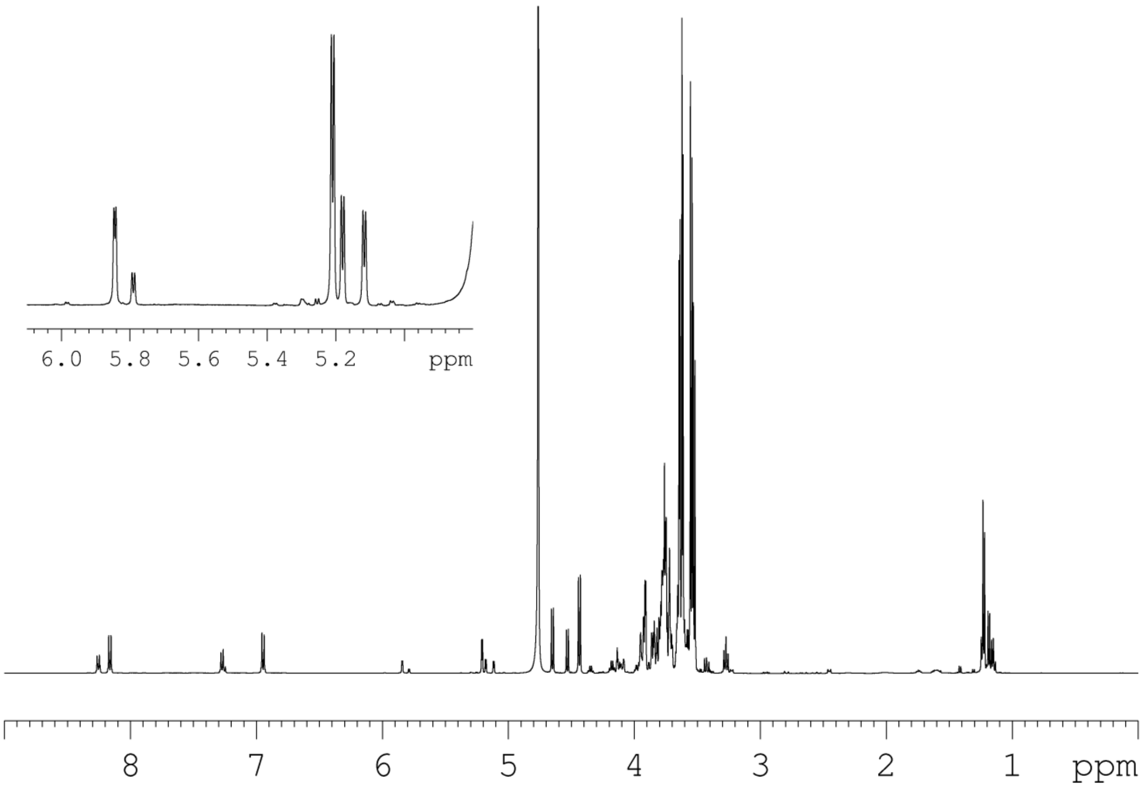

Figure IX. ^{1}H NMR spectrum (D_2O, 500 MHz) of post-enzymatic reaction mixture by Thma enzyme with expansion of the α-anomeric region.

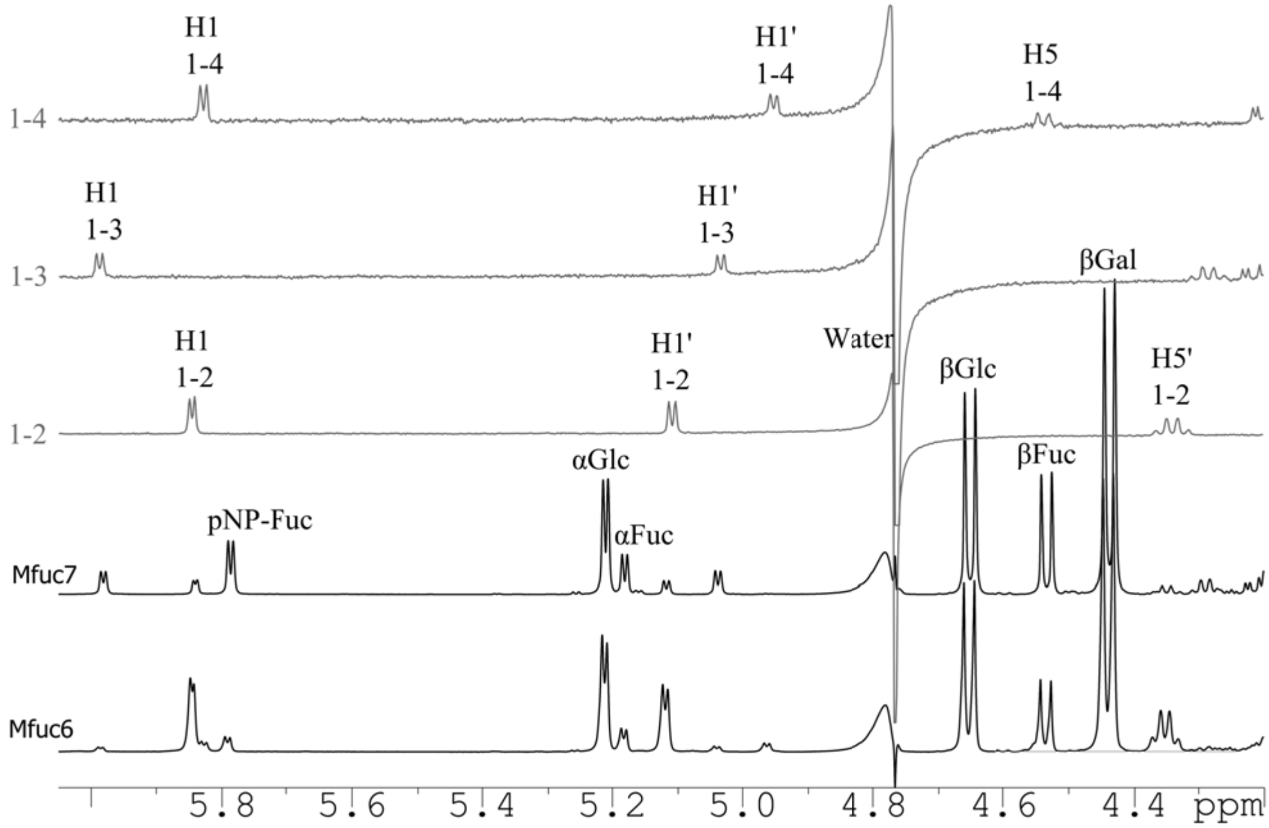

Figure X. ^{1}H NMR spectra (D_2O, 400 MHz) of individual self-condensation products separated by HPLC and comparison with products of selected enzymatic reactions. From the top: *p*-nitrophenyl α-L-fucopyranosyl-(1-4)-α-L-fucopyranoside, *p*-nitrophenyl α-L-fucopyranosyl-(1-3)-α-L-fucopyranoside,, *p*-nitrophenyl α-L-fucopyranosyl-(1-2)-α-L-fucopyranoside, reaction catalyzed by Mfuc7, and reaction catalyzed by Mfuc6. The residual solvent signals were (partially) removed by presaturation.

NMR chemical shifts of selected compounds characterized

p-nitrophenyl α-L-fucosyl-(1-2)-α-L-fucose

^{1}H NMR: δ 8.26 (d, 2H, *J* = 8.8 Hz, H3”), 7.28 (d, 2H, *J* = 8.8 Hz, H2”), 5.84 (d, 1H, *J* = 2.8 Hz, H1), 5.11 (d, 1H, *J* = 3.9 Hz, H1’), 4.34 (br. q, 1H, *J* = 6.5 Hz, H5’), 4.14 (m, 1H, H2),4.13 (m, 1H, H5), 4.11 (m, 1H, H3), 4.08 (m, 1H, H4), 3.97 (dd, 1H, *J* = 3.5 Hz, 10.5 Hz, H3’), 3.83 (m, 1H, H4’), 3.82 (m, 1H, H2’), 1.23 (d, 3H, *J* = 6.5 Hz, H6’), 1.15 (d, 3H, *J* = 6.7 Hz, H6). ^{13}C NMR derived from gHSQC and gHMBC: δ 162.2 (C1”), 143.0 (C4”), 126.9 (2*C3”), 117.6 (2*C2”), 97.6 (C1), 96.3 (C1’), 75.3 (C2), 72.7 (C4’), 70.3 (C3’), 68.7 (C4), 68.8 (C2’), 68.6 (C3), 67.9 (C5’), 66.7 (C5), 16.3 (C6’), 16.2 (C6).

p-nitrophenyl α-L-fucosyl-(1-3)-α-L-fucose

^{1}H NMR: δ 8.26 (d, 2H, *J* = 9.0 Hz, H3”), 7.28 (d, 2H, *J* = 9.0 Hz, H2”), 5.98 (d, 1H, *J* = 3.5 Hz, H1), 5.04 (d, 1H, *J* = 4.0 Hz, H1’), 4.28 (br. q, 1H, *J* = 6.7 Hz, H5’), 4.21 (dd, 1H, *J* = 3.4 Hz, 10.4 Hz, H3), 4.14 (br. q, 1H, *J* = 6.7 Hz, H5), 4.04 (dd, 1H, *J* = 3.5 Hz, 10.4 Hz, H2), 3.92 (m, 1H, H3’), 3.91 (m, 1H, H4), 3.80 (br. d, 1H, *J* = 3.3 Hz, H4’), 3.72 (dd, 1H, *J* = 4.0 Hz, 10.2 Hz, H2’), 1.21 (d, 3H, *J* = 6.7 Hz, H6’), 1.15 (d, 3H, *J* = 6.7 Hz, H6). ^{13}C NMR derived from gHSQC and gHMBC: δ 162.5 (C1”), 143.2 (C4”), 126.9 (2*C3”), 117.7 (2*C2”), 97.0 (C1’), 95.1 (C1), 72.7 (C2), 72.5 (C4), 72.3 (C4’), 70.1 (C3’), 68.8 (C3), 68.6 (C2’), 67.9 (C5’), 66.7 (C5), 16.3 (C6’), 16.1 (C6).

p-nitrophenyl α-L-fucosyl-(1-4)-α-L-fucose

^{1}H NMR: δ 8.26 (d, 2H, *J* = 9.2 Hz, H3”), 7.27 (d, 2H, *J* = 9.2 Hz, H2”), 5.83 (d, 1H, *J* = 3.9 Hz, H1), 4.95 (d, 1H, *J* = 4.0 Hz, H1’), 4.54 (br. q, 1H, *J* = 6.6 Hz, H5), 4.20 (m, 1H, H3), 4.18 (m, 1H, H5’), 4.04 (dd, 1H, *J* = 3.9 Hz, 10.6 Hz, H2), 3.94 (dd, 1H, *J* = 3.0 Hz, 10.8 Hz, H3’), 3.90 (br. d,

1H, *J* = 3.0 Hz, H4’), 3.83 (br. d, 1H, *J* = 3.0 Hz, H4), 3.80 (dd, 1H, *J* = 4.0 Hz, 10.8 Hz, H2’), 1.23 (d, 3H, *J* ~ 7 Hz, H6’), 1.17 (d, 3H, *J* = 6.6 Hz, H6). ^{13}C NMR derived from gHSQC and gHMBC: δ 162.3 (C1”), 143.1 (C4”), 126.8 (2*C3”), 117.6 (2*C2”), 101.3 (C1’), 97.7 (C1), 80.5 (C4’), 70.2 (C3’), 69.7 (C3), 69.5 (C2’), 69.5 (C5’), 68.8 (C4), 68.4 (C2), 67.7 (C5), 16.2 (C6’), 16.2 (C6).

Figure 7. NMR chemical shifts of selected compounds characterized in this study.

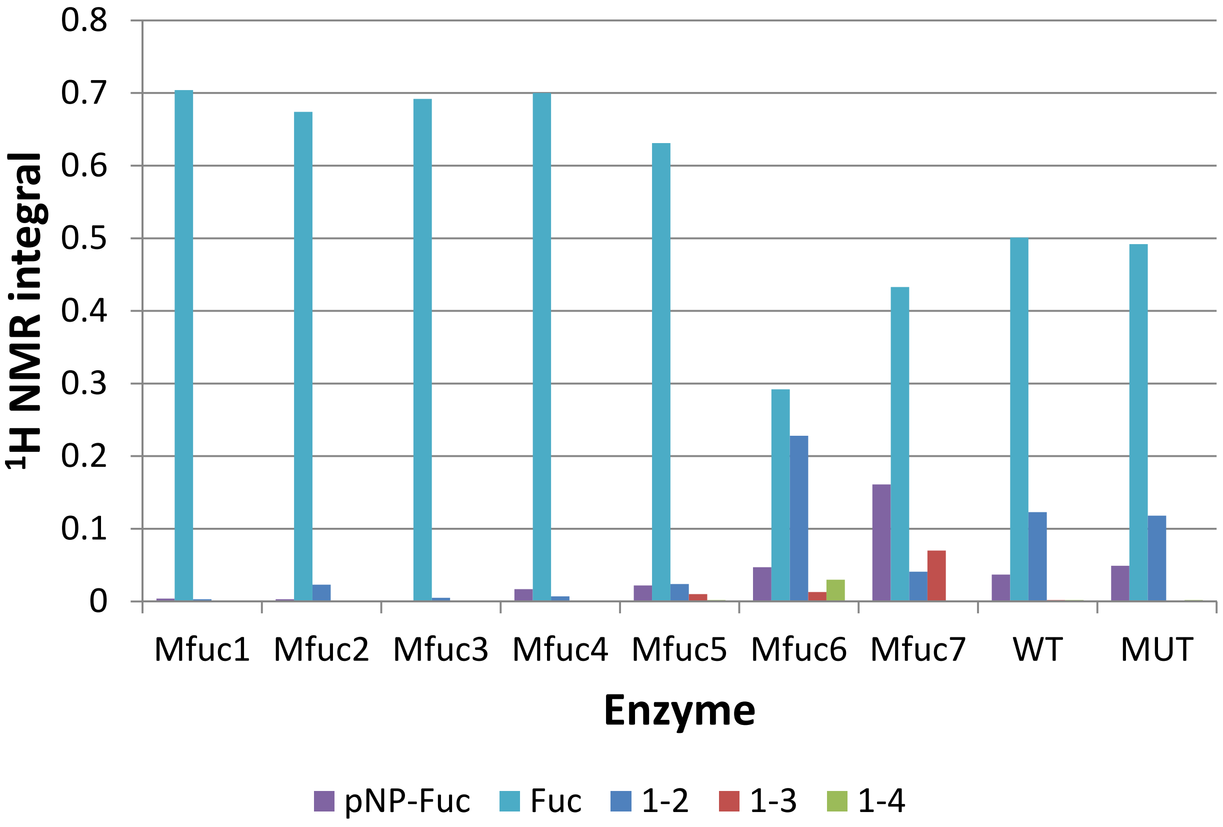

Figure XI. Comparison of ^{1}H NMR integrals for reaction mixtures of the different enzymes. Representative integrals of selected molecules were used to compare relative amounts within the NMR samples. For all samples the Gal anomeric resonance in lactose was calibrated to 1, which enables some comparison between samples, however with relatively high uncertainty. Anomeric resonances were used for integration. For the Fuc-Fuc-pNP products (1-2-, 1-3-, and 1-4-linked) the anomeric signal at the reducing end was used, for Fuc, the α- and β-anomeric resonances were added together.
