# Supplementary material for: Novel α-L-Fucosidases from a Soil Metagenome for Production of Fucosylated Human Milk Oligosaccharides
Source: PLoS One. 2016 Jan 22;11(1):e0147438. doi: 10.1371/journal.pone.0147438 (PMC4723247; doi:10.1371/journal.pone.0147438)
Supplement: S1 Table — (PDF) [file pone.0147438.s010.pdf]

S1 Table. List of primers used for amplification of α-L-fucosidase-encoding genes. Restriction sites are underlined, start and stop codons are indicated in bold.

Name	Sequence	Restriction site
mfuc1_fw	TAGCGGTCTCAC**ATG**TTGAAACCCAAACCGACCACC	*Bsa*I
mfuc1_rv	TAGCGGATCC**TTA**CTTCAAAGTCAACTCAATCACC	*Bam*HI
mfuc2_fw	TAGCGGTCTCAC**ATG**CAACACCAAGCGGCAG	*Bsa*I
mfuc2_rv	TAGCGGATCC**TTA**CTTCAGGAACAGCTCGATCACTG	*Bam*HI
mfuc3_fw	TAGCGGTCTCAC**ATG**GAAACACTTCCCTTTAGACAGGTC	*Bsa*I
mfuc3_rv	TAGCGGATCC**TTA**TTCTCTAGCGGCGCCCTG	*Bam*HI
mfuc4_fw	TAGCGGTCTCAC**ATG**TACACACCCAAACCGACGC	*Bsa*I
mfuc4_rv	TAGCGAATTC**CTA**CTCTTTCAGCTCCAGCTCGATG	*Eco*RI
mfuc5_fw	TAGCGGTCTCAC**ATG**AATAACGATCGAATGCAGTGG	*Bsa*I
mfuc5_rv	TAGCGAATTC**TCA**TTTCAACACCAGCTCCACG	*Eco*RI
mfuc6_fw	TAGCGGTCTCAC**ATG**AAAAAAACCAACACCTGGTTCG	*Bsa*I
mfuc6_rv	TAGCGAATTC**TCA**AGCCACGTCGATCGC	*Eco*RI
mfuc7_fw	TAGCGAAGACACC**ATG**ACAAGCTATGTGACCCGCAAAAC	*Bbs*I
mfuc7_rv	TAGCGAATTC**TTA**CTTCAGAAAAAGCTCAATGACCG	*Eco*RI
Thma_fw	ATCGGGTCTCCCATGATTTCAATGAAACCGCGTTATAAACC	*Bsa*I
Thma_rv	CGGGGTACCTTATTCCTCGACTGCTTCCAGAACC	*Kpn*I
