# Supplementary material for: Novel α-L-Fucosidases from a Soil Metagenome for Production of Fucosylated Human Milk Oligosaccharides
Source: PLoS One. 2016 Jan 22;11(1):e0147438. doi: 10.1371/journal.pone.0147438 (PMC4723247; doi:10.1371/journal.pone.0147438)
Supplement: S2 Table — (PDF) [file pone.0147438.s011.pdf]

S2 Table. Substrate specificity of characterised GH29 α-L-fucosidases. Abbreviations used as well as relevant references are listed below. The table is adapted from http://www.cazy.org/GH29_characterized.html.

Organism	Protein Name	EC#	GenBank	Uniprot	Reported substrates hydrolyzed	Reported substrates not hydrolyzed
Sulfolobus solfataricus P2	α-L-fucosidase (FucA1;SSO11867 + SSO3060)	3.2.1.51	AAK43159.1<br>AAK43160.1	Q97UG1<br>Q97UG2	pNP-Fuc, α-L-Fuc-(1-3)- α -L-Fuc-O-4-NP [1]	***NA***
Bacteroides thetaiotaomicron VPI-5482	α-1,3/1,4-L-fucosidase / α-galactosidase (BT2192;BT_2192)	3.2.1.111	AAO77299.1	Q8A5P6	3-FL, Le^a, Le^b, Le^x, Le^y,[2]	pNP-Fuc (very low), 2-FL (very low), Fucα1-3GlcNAc,Fucα1-4GlcNAc,Fucα1-6GlcNAc [2]
Bacteroides thetaiotaomicron VPI-5482	α-L-fucosidase (Fuc2970;BtFuc2970;BT2970;BT_2970)	3.2.1.51	AAO78076.1<br>NP_811882.1	Q8A3I4	pNP-Fuc, Fucα1-3GlcNAc, Fucα1-4GlcNAc (low) [2]	Fucα1-6GlcNAc, 2'FL, 3-FL, Le^a, Le^b, Le^x, Le^y [2]
Bifidobacterium bifidum JCM 1254	α-1,3/4-L-fucosidase (AfcB;BbAfcB)	3.2.1.111	BAH80310.1	C5NS94	3FL, Le^a, Le^b, Le^x, Le^y, LNFP II, LNFP III [3]	2'-FL, Fucα1-6GlcNAc, SLe^x (trace), pNP-Fuc [3]
Bifidobacterium longum subsp. *infantis* ATCC 15697	α-1,3/1,4-L-fucosidase (Blon_2336)	3.2.1.51<br>3.2.1.111	ACJ53394.1	B7GNN8	CNP-fucose, 3'FL, LNFP III, Le^a, Le^x, partially LNFP I [4]	2'-FL, Fucα1-2Gal [4]
Bifidobacterium longum subsp. *infantis* ATCC 15697	α-L-fucosidase (Blon_0248)	3.2.1.51	ACJ51376.1	B7GTT5	CNP-fucose, LNFP III, partially LNFP I [4]	2'-FL, 3-FL, Fucα1-2Gal [4]
Bifidobacterium longum subsp. *infantis* ATCC 15697	α-L-fucosidase (Blon_0426)	3.2.1.51	ACJ51546.1	B7GN40	CNP-fucose, LNFP III, partially LNFP I [4]	2'-FL, 3-FL, Fucα1-2Gal [4]
Lactobacillus casei BL23	α-L-fucosidase A (AlfA;LCABL_20390) (probable fragment)	3.2.1.51	CAQ67115.1	B3W8U6	pNP-Fuc, Fucα1-6GlcNAc (low) [5]	Fucα1-2Gal, Fucα1-2Galβ1-4GlcNAc, Fucα1-2Glc, Le^x, Le^a, Le^b, Fucα1-3GlcNAc, Fucα1-4GlcNAc [5]
Lactobacillus casei BL23	α-L-fucosidase B (AlfB;LCABL_28270)	3.2.1.51	CAQ67877.1	B3WB08	pNP-Fuc, Fucα1-3GlcNAc (high), Fucα1-2Gal, Fucα1-2Galβ1-4GlcNAc, 2'-FL, Fucα1-4GlcNAc [5]	3-FL, Le^x, Le^a, Le^b, Fucα1-6GlcNAc [5]
Lactobacillus casei BL23	α-L-fucosidase C (AlfC;LCABL_29340) (possible fragment)	3.2.1.51	CAQ67984.1	B3WBB5	pNP-Fuc, Fucα1-6GlcNAc (high), Fucα1-2Gal (low), Fucα1-3GlcNAc (low), Fucα1-4GlcNAc (low) [5]	Fucα1-2Galβ1-4GlcNAc, 2'FL, 3-FL, Le^x, Le^a, Le^b [5]

Paenibacillus thiaminolyticus	α-L-fucosidase (aLfuk1)	3.2.1.51	CBM40947.1	E3PQQ9	pNP-fuc [6]	***NA***
Streptomyces sp. 142	α-1,3/1,4-L-fucosidase (N/A)	3.2.1.111	AAD10477.1	Q9Z4I9	Oligosaccharides labeled with 2-amionpyridine:<br><br>LNFPII-PA, LNFPIII-PA, Fucα1-2LNFPII-PA	Oligosaccharides labeled with 2-amionpyridine<br><br>sialylated-LNFPII-PA (very low), 2'-FL-PA (very low), LNFPI-PA, …
Tannerella forsythia ATCC 43037	α-1,2-L-fucosidase (Fuc1;TfFuc1;BFO_2737)	3.2.1.63	AEW21393.1	G8UMQ6	pNP-fuc, 2'FL H-trisaccharide, Fucα1-6GlcNAc [7]	3-FL, Le^a, A antigen tetraose,<br><br>Fuc(α1-4)Gal [7]
Thermotoga maritima MSB8	α-L-fucosidase (TM0306)	3.2.1.51	AAD35394.1	Q9WYE2	pNP-fuc [8]	***NA***
Xanthomonas campestris pv. *campestris* ATCC 33913	[N-glycan] α-1,3-L-fucosidase (NixE;FucA1;XCC2888)	3.2.1.51	AAM42160.1	Q8P6S7	AMC (7-amino-4-methylcoumarin) substrates:<br><br>LNFPII-AMC, LNFPIII-AMC [9]	AMC (7-amino-4-methylcoumarin) substrates:<br><br>2'-FL-AMC [9]
Arabidopsis thaliana	α-1,3/4-L-fucosidase 1 (AtFUC1;At2g28100)	3.2.1.111	NP_180377.2	Q8GW72<br>Q9ZUV2	2-aminobenzamide labeled LNFP II, 3FL, fucose linked α1-4 to non-reducing end in higher DP oligoes [10]	2-aminobenzamide labeled 2'-FL, pNP-Fuc, fucose linked α1-3 to reducing end [10]
Canis lupus familiaris	α-L-fucosidase (FUCA1)	3.2.1.51	CAA63362.1	P48300	4-methylumbelliferyl-α-L-fucopyranoside [11]	***NA***
Dictyostelium discoideum	α-L-fucosidase (AlfA)	3.2.1.51	AAO51149.1	P10901	6-O-alpha-L-fucopyranosyl-N-acetylglucosaminitol, low activity on 2'-fucosyllactitol, very low on 3-fucosyllactitol and Lacto-N-fucopentaitol I [12]	Lacto-N-fucopentaitol II, Lacto-N-fucopentaitol III [12]
Drosophila melanogaster	α-L-fucosidase (Fuca;DmFuca;CG6128)	3.2.1.51	AAM50292.1	Q9VTJ4	***NA***	***NA***
Fusarium graminearum PH-1	α-L-fucosidase (Fco1;FgFCO1;fg11254)	3.2.1.51	AFR68935.1	J9UN47	pea XG oligosaccharides (Fucα1-2Galβ1-2Xyl in XXFG) [13], 2'-FL, Fucα1-2Gal (low) [14]	pNP-Fuc (very weak), 3-FL, Le^a, Le^x (very weak) [14]

Fusarium oxysporum 0685	α-L- fucosidase (Fco1;FoFCO1)	3.2.1.51	AFR68934.1	J9TMS7	pNP-Fuc [13], fucoidan, porcine gastrin mucin [15]	***NA***
Homo sapiens	α-L-fucosidase (FucA1)	3.2.1.51	AAA52481.1	P04066 Q8NAC2	4-methylumbelliferyl α-L-fucopyranoside, pNP-Fuc, 2'-FL, LNFPII, LNFPIII, α1-2Fuc-decasaccharide, Fucα1-6GlcNAc, α-L-fucose residues linked to the 2 position of galactose or the 3, 4, or 6 position of N-acetylglucosamine [16]	4-methylumbelliferyl β-fucopyranoside, 4-methylumbelliferyl α-galactopyranoside, pNP-β-Fuc [16]
Homo sapiens	α-L-fucosidase (FucA2;Mgc1314;dJ20N2.5)	3.2.1.51	CAB53746.1	Q7Z6V2 Q9BTY2	Hydrolyzes pNP-Fuc, prefers α1-2 but capable of cleaving some α1-3, and α1-4 linkages [17]	***NA***
Rattus norvegicus	α-L-fucosidase (FucA1;FucA)	3.2.1.51	CAA34268.1	P17164	Hydrolyzes pNP-Fuc, α1-2, α1-3, and α1-4 fucosyl linkages, and glycopeptides derived from β-glucoronidase but not native glycopeptides, lower activity on α1-6 linkages [18]	Fucα1-2GlcNAcβ-pNP

Abbreviations used:

2'FL	Fucα1-2Galβ1-4Glc
3-FL	Galβ1-4(Fucα1-3)Glc
A antigen tetraose	GalNAcβ1-3(αFuc1-2)Galβ1-4Glc
Antigen H disaccharide	Fucα1-2Galβ
Antigen H type II trisaccharide	Fucα1-2Galβ1-4GlcNAc
CNP-Fucose	2-chloro-4-nitrophenyl-α-L-fucopyranoside
Le^a trisaccharide	Galβ1-3(Fucα1-4)GlcNAc
Le^b trisaccharide	Fucα1-2Galβ1-3(Fucα1-4)GlcNAc
Le^x trisaccharide	Galβ1-3(Fucα1-3)GlcNAc
Le^y trisaccharide	Fucα1-2Galβ1-3(Fucα1-3)GlcNAc
LNFP I	Fucα1-2Galβ1-3GlcNAcβ1-3Galβ1-4Glc
LNFPII	Galβ1-3(Fucα1-4)GlcNAcβ1-3Galβ1-4Glc
LNFPIII	Galβ1-3(Fucα1-3)GlcNAcβ1-3Galβ1-4Glc
LNFPV	Galβ1-3GlcNAcβ1-3Galβ1-4(Fucα1-3)Glc
pNP-Fuc	4-Nitrophenyl α-L-fucopyranoside

References

1. Cobucci-Ponzano B, Trincone A, Giordano A, Rossi M, Moracci M. Identification of an Archaeal α-l-Fucosidase Encoded by an Interrupted Gene. J Biol Chem. 2003;278: 14622–14631.
2. Sakurama H, Tsutsumi E, Ashida H, Katayama T, Yamamoto K, Kumagai H. Differences in the Substrate Specificities and Active-Site Structures of Two - L -Fucosidases (Glycoside Hydrolase Family 29) from Bacteroides thetaiotaomicron. Biosc Biotech Biochem. 2012;76: 1022–1024.
3. Ashida H, Miyake A, Kiyohara M, Wada J, Yoshida E, Kumagai H, et al. Two distinct ??-L-fucosidases from Bifidobacterium bifidum are essential for the utilization of fucosylated milk oligosaccharides and glycoconjugates. Glycobiology. 2009;19: 1010–1017.
4. Sela D a., Garrido D, Lerno L, Wu S, Tan K, Eom HJ, et al. Bifidobacterium longum subsp. infantis ATCC 15697 α-fucosidases are active on fucosylated human milk oligosaccharides. Appl Environ Microbiol. 2012;78: 795–803.
5. Rodríguez-Díaz J, Monedero V, Yebra MJ. Utilization of natural fucosylated oligosaccharides by three novel α-L-fucosidases from a probiotic lactobacillus casei strain. Appl Environ Microbiol. 2011;77: 703–705.
6. Benesová E, Lipovová P, Dvoráková H, Králová B. α-L-fucosidase from Paenibacillus thiaminolyticus: its hydrolytic and transglycosylation abilities. Glycobiology. 2013;23: 1052–65.
7. Megson ZA, Koerdt A, Schuster H, Ludwig R, Janesch B, Frey A, et al. Characterization of an α-l-fucosidase from the periodontal pathogen Tannerella forsythia. Virulence. 2015;6: 282–92.
8. Osanjo G, Dion M, Drone J, Solleux C, Tran V, Rabiller C, et al. Directed Evolution of the R- L -Fucosidase from Thermotoga maritima into an. Biochemistry. 2007;46: 1022–1033.
9. Wong-Madden ST, Landry D. Purification and characterization of novel glycosidases from the bacterial genus Xanthomonas. Glycobiology. 1995;5: 19–28.
10. Zeleny R, Leonard R, Dorfner G, Dalik T, Kolarich D, Altmann F. Molecular cloning and characterization of a plant α1,3/4-fucosidase based on sequence tags from almond fucosidase I. Phytochemistry. 2006;67: 641–648.
11. Bielicki J, Muller V, Fuller M, Hopwood JJ, Anson DS. Recombinant canine alpha-l-fucosidase: expression, purification, and characterization. Mol

Genet Metab. 2000;69: 24–32.

12. Schopohl D, Müller-Taubenberger A, Orthen B, Hess H, Reutter W. Purification and properties of a secreted and developmentally regulated alpha-L-fucosidase from Dictyostelium discoideum. J Biol Chem . 1992;267 : 2400–2405.

13. Paper JM, Scott-Craig JS, Cavalier D, Faik A, Wiemels RE, Borrusch MS, et al. α-Fucosidases with different substrate specificities from two species of Fusarium. Appl Microbiol Biotechnol. 2012;97: 5371–80.

14. Cao H, Walton JD, Brumm P, Phillips GN. Structure and substrate specificity of a eukaryotic fucosidase from Fusarium graminearum. J Biol Chem. 2014; 0–31.

15. Yamamoto K, Tsuji Y, Kumagai H, Tochikura T. Induction and Purification of α-l-Fucosidase from Fusarium oxysporum. Agric Biol Chem. Taylor & Francis; 1986;50: 1689–1695.

16. Dawson G, Tsay G. Substrate specificity of human alpha-L-fucosidase. Arch Biochem Biophys. UNITED STATES; 1977;184: 12–23.

17. Dicioccio RA, Barlow JJ, Khushi L. Substrate Specificity and Other Properties of a-L-Fucosidase from Human Serum *. J Biol Chem. 1982;257: 714–718.

18. Opheim DJ, Touster O. Complex Carbohydrates Part C [Internet]. Methods in Enzymology. Elsevier; 1978.
